# A comprehensive linkage map and QTL map for carcass traits in a cross between Giant Grey and New Zealand White rabbits

**DOI:** 10.1186/s12863-015-0168-1

**Published:** 2015-02-11

**Authors:** Ina Sternstein, Monika Reissmann, Dorota Maj, Josef Bieniek, Gudrun A Brockmann

**Affiliations:** Department for Crop and Animal Sciences, Breeding Biology and Molecular Genetics, Faculty of Live Science, Humboldt-Universität zu Berlin, Invalidenstr. 42, 10115 Berlin, Germany; Department of Genetics and Animal Breeding, Agricultural University of Kraków, Al. Mickiewicza 24/28, 30-059 Kraków, Poland

**Keywords:** Linkage map, QTL, Carcass composition, Meat quality, Rabbit

## Abstract

**Background:**

Genomic resources for the rabbit are still limited compared to many other livestock species. The genomic sequence as well as linkage maps have gaps that hamper their use in rabbit genome research. Therefore, the aims of this study were the improvement of existing linkage maps and the mapping of quantitative trait loci (QTL) for carcass and meat quality traits. The study was performed in a F_2_ population of an initial cross between Giant Grey (GG) and New Zealand White (NZW) rabbits. The population consisted of 363 F_2_ animals derived from 9 F_1_ bucks and 33 F_1_ does. 186 microsatellite and three SNP markers were informative for mapping.

**Results:**

Out of 189 markers, which could be assigned to linkage groups, 110 markers were genetically mapped for the first time. The average marker distance was 7.8 cM. The map across all autosomes reached a total length of 1419 cM. The maternal linkage map was 1.4 times longer than the paternal. All linkage groups could be anchored to chromosomes. On the basis of the generated genetic map, we identified a highly significant QTL (genome-wide significance p < 0.01) for different carcass weights on chromosome 7 with a peak position at 91 cM (157 Mb), a significant QTL (p < 0.05) for bone mass on chromosome 9 at 61 cM (65 Mb), and another one for drip loss on chromosome 12 at 94 cM (128 Mb). Additional suggestive QTL were found on almost all chromosomes. Several genomic loci affecting the fore, intermediate and hind parts of the carcass were identified. The identified QTL explain between 2.5 to 14.6% of the phenotypic variance in the F_2_ population.

**Conclusions:**

The results present the most comprehensive genetic map and the first genome-wide QTL mapping study for carcass and meat quality traits in rabbits. The identified QTL, in particular the major QTL on chromosome 7, provide starting points for fine mapping and candidate gene search. The data contribute to linking physical and genetic information in the rabbit genome.

**Electronic supplementary material:**

The online version of this article (doi:10.1186/s12863-015-0168-1) contains supplementary material, which is available to authorized users.

## Background

Rabbit meat is healthy and in many countries a delicious protein source for human nutrition. As such, carcass composition and meat quality are of economic importance for rabbit breeders. The effective improvement of breeding requires the understanding of the genomic architecture and genomic information of such complex traits. Compared to other farm animal species, genomic resources for the rabbit are still limited. Although the rabbit genome has been sequenced (http://www.ensembl.org/Oryctolagus_cuniculus/, Ensembl 73, OryCun 2.0), currently only about 82% of the 2.74 Gigabase of the rabbit genome have been anchored to chromosomes. The existing microsatellite based linkage maps for the rabbit were built in two reference populations, one at INRA (France) using three rabbit INRA strains (INRA2066, Castor Orylag and Laghmere, [1]) and the other at the Utrecht University (Netherlands) using an F_2_ intercross of the rabbit strains AX/JU and IIIVO/JU [2,3]. These maps do not cover all rabbit chromosomes.

A comprehensive linkage map can help to improve the annotation and sequence assembly of the rabbit genome since it can link existing sequences of non-anchored rabbit bacterial artificial chromosomes (BACs) to the genome assembly. Such a genetic map is also an essential condition for the mapping of QTL in structured pedigrees which is an important step in quantitative trait gene identification. Even in the era of genome-wide association studies (GWAS), which are performed in unstructured populations, linkage mapping in families provides reliable genomic positions which support accurate mapping in GWAS.

This study aimed at building a comprehensive linkage map, which is anchored to the existing physical map, and using this information for mapping QTL for carcass and meat quality traits. The study was performed in an F_2_ pedigree of a cross between GG and NZW rabbits.

## Results and discussion

### Pedigree specific linkage map

Out of 387 known microsatellites [2-10], which were initially tested (Additional file 1: Table S1), 186 microsatellite and additionally three SNP markers in the *myostatin* (*MSTN*) gene [11], were informative for the cross between GG and NZW. The myostatin gene is located on OCU7 at 130,429,151 bp (http://www.ensembl.org/Oryctolagus_cuniculus/, Ensembl 73, OryCun 2.0). The physical position corresponds to 75.9 cM in the generated genetic map. The gene resides between the markers D7Utr3 und D7Utr4 that we used in this study. The number of observed alleles for each of the 186 microsatellites varied from two to eight with an average of 3.2 ± 1.0 in the founder animals (Additional file 2: Table S2). The heterozygosity index for all informative markers in the F_1_ population ranged from 0.13 to 1.00 with an average of 0.65 ± 0.22. For the number of informative meioses a variation between 54 and 775 (468.6 ± 177.6) was observed, among these 0 to 558 meioses (158.3 ± 129.9) had known phases. The mean polymorphism information content per marker in the F_2_ population was 0.43 ± 0.14 and varied from 0.09 (INRACCDDV0074, INRACCDDV0017) to 0.77 (INRACCDDV0293, data not shown).

The 186 microsatellite and three SNP markers could be assigned to 21 linkage groups. Twenty linkage groups are located on *Oryctolagus cuniculus* (OCU) chromosomes 1 to 19 and X. Two linkage groups were assigned to OCU4, but could not be linked to each other (Additional file 3: Figure S1). Out of 189 markers, 110 markers were genetically mapped for the first time. For 53 markers, we could confirm their cytogenetic positions. Compared to existing linkage groups, which were generated in other crosses, the cytogenetic positions could not be confirmed for five markers (INRACCDDV0084, INRACCDDV0218, INRACCDDV0219, INRACCDDV0230, INRACCDDV0256) in our cross. Twelve markers, which had sequence information, but had not been previously mapped, neither cytogenetically, genetically nor physically, were mapped in our population for the first time. These markers are INRACCDDV0103, INRACCDDV0165, INRACCDDV0194, INRACCDDV0302, D5L1C3, D6L2B5, D6L2H3, D6L3H10, D12L1C2, D12L1E11, D12L4A1 and OCRLADF4. These markers are particularly valuable for the mapping of BAC clones to the genome assembly to improve the rabbit genomic sequence (Additional file 2: Table S2) [1-4,6-8,10,12-17].

Among 161 markers with known physical position (http://www.ensembl.org/Oryctolagus_cuniculus/, Ensembl 73, OryCun 2.0), 155 markers had consistent positions in the genetic and physical maps. Six markers could not be mapped to their expected genomic positions. Although they were assigned to linkage groups on the expected chromosome (OCU6, 7, 8, 15 and 18) they had different positions on the chromosome. The affected markers are D6Utr4 (OCU6: 25.038137), D7L2F2 (OCU7: 60.680119), INRACCDD0323 (OCU7: 59.306464), INRACCDDV0080 (OCU8: 37.478525), INRACCDDV0143 (OCU15: 108.340756) and INRACCDDV0218 (OCU18: 68.515123). The position of the marker INRACCDDV0143 is inconsistent with respect to different published cytogenetic positions [1,4]. The map position identified in our population corresponds with the cytogenetic position 15q12 [1].

Seven other microsatellite markers, for which a cytogenetic position was reported [1,4,12,14], could not be linked to neighbouring markers in the expected region, instead they showed a close linkage to loci on other chromosomes in our cross. This refers to markers INRACCDDV0230 (14p11) [4] and INRACCDDV0219 (6p12-p13) [12], which were assigned to OCU13, markers INRACCDDV0218 (OCU3q14) [12] and INRACCDDV0256 (OCUXq12prox) [4], which were relocated to OCU18, and markers INRACCDDV0213 (OCU6p12prox) [1,12], INRACCDDV0127 (OCU6) [1] and INRACCDDV0084 (OCU20q12) [14], which showed an X-linked inheritance in our population. The new marker positions were confirmed by sequence alignments to the rabbit genome (http://www.ensembl.org/Oryctolagus_cuniculus/, Ensembl 73, OryCun 2.0).

The calculated order of the other markers in the corresponding linkage groups on chromosomes 1, 3, 5, 7, 8, 11, 14, 16 and 19 is consistent with previous maps [1-3], although, slight differences with regard to the distances between markers occur. This is consistent with our knowledge about pedigree specific linkage maps [18,19]. Differences in the marker order between our map and previously published maps were identified for linkage groups on chromosomes 4, 6, 9, 13, 15 and 18. Because different markers were used in different mapping populations for chromosomes 2, 10, 12, 17, 20, 21 and X, maps cannot be compared.

Since different resource populations were used to construct linkage maps, deviations in marker distances, marker orders and even positions on different chromosomes can be expected. While mapping errors cannot be excluded entirely, different mapping positions could mainly result from genome reorganizations between the different breeds that were used in the generation of resource populations for mapping the markers. The information about deviant marker positions in different populations is valuable for the genomic assembly of the rabbit genome sequence as well as for genetic and maybe even phenotypic diversity.

Maps calculated from maternal meioses across all autosomes were on average 1.4 times longer than paternal maps. Higher recombination rates in females are also consistent with findings in other species, for example in pigs [20], cattle [21], humans [22], and mice [23]. However, in distinct regions on OCU4 (LG4b), OCU9 and OCU16 maternal maps were shorter than paternal maps, a result that was also observed in pigs, e. g. [18,19]. The ratios of genetic lengths between the female and male maps varied from 0.7 for OCU4 (LG 4b) and OCU16 to 5.4 for OCU11.

With a total genetic length for all autosomes of 1419 cM and an average marker distance of 7.8 cM our genetic map provides the linkage map with the highest marker coverage. Nevertheless, exceptions to good coverage still exist on chromosomes 20, 21 and Y, because no marker mapped to OCU20 and OCUY, and only one marker could be assigned to OCU21.

### Phenotypic characteristics and correlation between traits

GG rabbits have about 500 g higher liveweights than NZW rabbits at the age of 84 days (Table 1). Since the total body, carcass and meat weights as well as the portions of head, fore, intermediate and hind parts are important for rabbit breeders, we have analysed all these traits. For all carcass traits, GG rabbits have higher weights compared to NZW rabbits. The F_1_ and F_2_ means of hot and reference carcass weights shifted towards the mean value of the GG breed suggesting dominance components in the mode of inheritance. The weight of the intermediate part and the meat weights of fore and intermediate parts in F_1_ rabbits exceeded the average performances of their parents. As expected, the liveweight and carcass weights as well as the total weight and the meat weight of the different carcass parts were highly correlated (*r* ≥ 0.89, p < 0.0001, data not shown); bone weights and head weight showed high correlations to the other carcass traits (*r* ≥ 0.62, p < 0.0001, Table 2). Similar results for highly correlation between carcass traits were observed in other studies [24-26]. Drip loss of the whole carcass showed low correlation with the pH value 24 hours p.m. at M. *biceps femoris* (*r* = 0.19, p < 0.0001, Table 2). This is in line with the correlation (*r* = 0.20) found between the pH value of M. *biceps femoris* and water holding capacity (WHC) of M. *longissimus dorsi* in a three-way cross [27]. Most phenotypic correlations between carcass composition and meat quality parameters are low and partially negative (−0.28 ≤ *r* ≤ 0.31, p < 0.05, Table 2). Studies using principal component analysis indicated that all colour measurements, pH values and fat had low correlations [28,29]

**Table 1:** **Phenotypic characterisation of parental breeds, F**_**1**_ **and F**_**2**_ **animals of the cross between GG and NZW**

	**GG** ^**5**^	**NZW** ^**5**^	**F**_**1**_	**F**_**2**_
**Trait**	**Mean±SD**	**Mean±SD**	**Mean±SD**	**Mean±SD**
	**n = 4**	**n = 18**	**n = 21**	**n = 363**
Liveweight (g)	3048.75±255.81^a^	2522.56±126.72^b^	2647.29±269.31^b,c^	2644.43±498.53^a,c^
Hot carcass weight (g)	1426.50±175.64^a^	1213.33±83.24^b^	1357.10±132.42^a^	1344.46±271.79^a^
Reference carcass weight (g)	1385.75±178.58^a^	1174.50±77.42^b^	1301.95±127.51^a^	1304.09±265.55^a^
Fore part weight (g)	563.50±69.55^a^	462.78±38.13^b^	521.67±54.26^a^	523.44±111.66^a^
Intermediate part weight (g)	292.25±48.22^a,b^	264.39±20.21^a^	296.24±39.11^b,c^	289.89±65.55^b,c^
Hind part weight (g)	529.50±63.49^a,b^	446.89±28.41^a^	482.90±43.21^b,c^	490.59±94.81^b,c^
Meat weight fore part (g)^1^	402.50±63.57^a,b^	360.11±36.58^a^	400.67±44.06^b^	382.00±88.14^b^
Meat weight intermediate part (g)^1^	238.25±51.01^a^	227.50±15.98^a^	242.05±31.95^a^	230.42±51.66^a^
Meat weight hind part (g)^1^	414.75±49.85^a^	363.83±26.77^b^	390.86±37.05^a^	377.73±77.30^a,b^
Bone weight fore part (g)^1^	148.25±27.26^a,c^	98.50±8.06^b^	114.57±13.12^a^	125.54±27.00^c^
Bone weight intermediate part (g)^1^	41.25±5.91^a^	27.94±4.02^b^	34.43±5.87^c^	39.53±10.00^a^
Bone weight hind part (g)^1^	114.50±17.69^a^	83.11±6.01^b^	90.33±11.0^c^	101.73±22.03^a^
Head weight (g)^1^	180.25±6.80^a^	167.17±11.56^b^	163.10±10.52^b^	154.21±22.97^c^
Kidney weight (g)^1^	27.00±6.38^a^	17.28±1.60^a,c^	20.10±4.32^b^	17.43±3.76^c^
Scapular fat weight (g)^1^	2.18±1.13^a,b^	0.90±1.17^a^	1.19±1.09^b^	1.93±1.42^a^
Perirenal fat weight (g)^1^	4.39±1.94^a,bc^	3.19±2.82^a^	6.53±2.52^b^	4.84±2.82^c^
Inguinal fat weight (g)^1^	0.00±0.00^a^	0.04v0.16^a,b^	0.36±0.69^b^	1.10±1.13^c^
Drip loss (%)	2.91±0.57^a,b^	3.18±1.04^a,b^	4.05±1.73^b^	2.98±0.84^a,c^
pH_45_ value M. *biceps femoris*	6.99±0.42^a^	6.82±0.20^a^	6.44±0.24^b^	6.65±0.30^c^
pH_24_ value M. *biceps femoris*	5.79±0.25^a,c^	5.81±0.11^a,b^	5.61±0.11^b^	5.75±0.19^c^
Meat coulor_45_ L* M. *biceps femoris* ^2^	51.01±1.66^a^	-	55.48±1.16^b^	57.10±2.16^c^
Meat coulor_24_ L* M. *biceps femoris* ^2^	58.46±0.59^a^	-	56.15±1.31^b^	57.63±1.95^a^
Meat coulor_45_ a* M. *biceps femoris* ^2^	2.91±0.84^a^	-	12.19±1.08^b^	11.24±1.51^c^
Meat coulor_24_ a* M. *biceps femoris* ^2^	4.11±1.11^a^	-	14.06±0.98^b^	12.90±1.67^c^
Meat coulor_45_ b* M. *biceps femoris* ^2^	1.82±0.73^a^	-	1.10±1.19^a^	1.10±1.40^a^
Meat coulor_24_ b* M. *biceps femoris* ^2^	4.78±0.97^a,b^	-	4.67±0.85^a^	3.58±1.49^b^
Shear force^3^	-	-	3.11±0.90^a^	3.35±0.85^a^
Protein content (%)^4^	-	22.76±0.54^a^	23.22±0.48^b^	23.40±0.58^b^
Fat content (%)^4^	-	2.83±0.85^a^	0.65±0.33^b^	0.80±0.37^b^

^1^number of F_2_ animals = 327; ^2^number of F_2_ animals = 336; ^3^number of F_2_ animals = 155; ^4^number of F_2_ animals = 93; ^5^data for some meat quality traits were not recorded in the founder breeds; pH_45_-pH value 45 min *post mortem*, pH_24_-pH value 24 h *post mortem*, meat colour_45_ - meat colour 45 min *post mortem*, meat colour_24_- meat colour 24 h *post mortem*, L* - lightness, a*-redness, b*-yellowness; ^a,b,c^Significant differences between parental, F_1_ and F_2_ for the same trait (t-test, p < 0.05).

**Table 2:** **Pearson’s correlation coefficients between carcass composition and meat quality traits** ^**1**^

	**BW**	**BW**	**BW**	**SFa**	**PFa**	**IFa**	**HW**	**KiW**	**pH**_**45**_	**pH**_**24**_	**DL**	L*_45_	L*_24_	a*_45_	a*_24_	b*_45_	b*_24_	**Pr**	**Fa**
	**FP**	**IP**	**HP**	**W**	**W**	**W**			**BF**	**BF**		**BF**	**BF**	**BF**	**BF**	**BF**	**BF**	**LD**	**LD**
LW	**.80**	**.73**	**.77**	**.43**	**.44**	.18**	**.88**	**.61**	(−.04)	(−.11)	−.14*	(.14)	(.13)	−.15*	(−.08)	(−.10)	(−.10)	−.39**	(.01)
HCW	**.81**	**.72**	**.77**	**.44**	**.49**	**.23**	**.89**	**.58**	(−.03)	(−.09)	(−.12)	.15*	(.09)	−.15*	(−.07)	(−.10)	(−.14)	−.34**	(.00)
RCW	**.81**	**.72**	**.77**	**.44**	**.49**	**.23**	**.89**	**.58**	(−.04)	(−.10)	−.17*	.16*	(.09)	−.16*	(−.08)	(−.10)	−.15*	−.34**	(.00)
FPW	**.82**	**.68**	**.76**	**.44**	**.47**	**.22**	**.89**	**.58**	(−.04)	(−.10)	−.16*	(.13)	(.07)	(−.11)	(−.04)	(−.07)	−.14*	−.34**	(−.01)
IPW	**.68**	**.70**	**.66**	**.47**	**.62**	.20**	**.79**	**.57**	(−.05)	(−.08)	−.15*	(.12)	(.10)	−.16*	(−.08)	(−.10)	(−.10)	−.28*	(.03)
HPW	**.83**	**.74**	**.81**	**.39**	**.41**	**.25**	**.91**	**.55**	(−.03)	(−.09)	−.17*	.21**	(.10)	−.20**	(−.12)	(−.13)	−.18*	−.37**	(−.02)
MWFP	**.72**	**.61**	**.70**	**.40**	**.48**	.21**	**.87**	**.59**	(.00)	(−.09)	(−.12)	(.13)	(.07)	(−.10)	(−.02)	(−.06)	(−.10)	−.34**	(−.06)
MWIP	**.66**	**.62**	**.62**	**.46**	**.53**	.18*	**.77**	**.59**	(.01)	(−.04)	(−.13)	(.12)	(.11)	−.15*	(−.08)	(−.06)	(−.07)	−.36**	(−.07)
MWHP	**.78**	**.67**	**.71**	**.40**	**.42**	**.22**	**.89**	**.58**	(−.01)	(−.10)	−.16*	.21**	(.10)	−.18*	(−.10)	(−.10)	−.15*	−.40**	(−.08)
BWFP	1.0	**.74**	**.81**	**.25**	**.24**	**.23**	**.78**	**.46**	(−.03)	(−.08)	−.18*	.21**	(.10)	−.17*	−.18*	(−.13)	−.25**	−.40**	(−.11)
BWIP		1.0	**.78**	**.24**	**.25**	**.23**	**.67**	**.45**	(−.04)	−.17*	(−.13)	**.31**	.23	**−.27**	**−.28**	−.18*	−.24**	**−.44**	(.02)
BWHP			1.0	**.23**	.20*	(.09)	**.78**	**.38**	(.03)	(−.06)	(−.10)	.20**	(.13)	−.22**	−.24**	−.16*	−.25**	−.39**	(−.06)
SFaW				1.0	**.43**	(.05)	**.33**	**.33**	(.02)	(.02)	(−.03)	(−.10)	(.05)	(−.06)	(−.03)	(.00)	(.02)	(−.08)	(.04)
PFaW					1.0	(.10)	**.31**	**.40**	−.15*	(−.11)	(−.07)	(−.13)	(.00)	(.10)	(.10)	.15*	(.09)	(.04)	(.08)
IFaW						1.0	**.30**	(.12)	(−.07)	(−.10)	(−.08)	.24**	(.05)	(−.12)	(−.07)	−.19	(−.13)	**−.43**	(.06)
HW							1.0	**.49**	(−.04)	(−.09)	−.15*	.23*	(.08)	(−.16)	(−.14)	−.19**	−.21**	**−.41**	(−.01)
KiW								1.0	(−.04)	(−.11)	(−.09)	(.03)	.18**	(−.03)	(.02)	(.02)	(.11)	−.30*	(.10)
pH_45_BF									1.0	**.39**	(.12)	(.08)	(.07)	(−.11)	(−.09)	.17*	(.14)	(−.09)	(−.02)
pH_24_BF										1.0	.19**	(−.03)	(.07)	(−.01)	(−.02)	.18**	.20**	(−.04)	(−.06)
DL											1.0	(−.12)	(−.05)	(.11)	.18**	(.09)	.24	.25*	(−.05)
L*_45_BF												1.0	.54	−.71	−.58	−.33	−.39	−.47	(.16)
L*_24_BF													1.0	−.54	−.65	−.26	−.14*	−.45**	(.19)
a*_45_BF														1.0	.74	.47	.40	.45	(−.23)
a*_24_BF															1.0	.33	.56	.40**	(−.07)
b*_45_BF																1.0	.36	.37**	(−.21)
b*_24_BF																	1.0	.35**	(.14)
PrLD																		1.0	−.30**

^1^levels of significance: bold values are significant at p < 0.0001; asterisks mark different significances *p < 0.01; **p < 0.001; values in parentheses are not significant. *Abbreviations: LW* live weight, *HCW* hot carcass weight, *RCW* reference carcass weight, *FPW* fore part weight, *IPW* intermediate part weight, *HPW* hind part weight, *MWFP* meat weight fore part, *MWIP* meat weight intermediate part, *MWHP* meat weight hind part, LD, M. *longissimus dorsi*, BF, M. *biceps femoris*, pH_45_ - pH value 45 min p.m.; pH_24_ - pH value 24 h p.m, L*_45_ and L*_24_, lightness 45 min and 24 h p.m.; a*_45_ and a*_24_, redness 45 min and 24 h p.m.; b*_45_ and b*_24_, yellowness 45 min and 24 h p.m.; DL, drip loss ; PrLD, protein content of M. *longissimus dorsi*; FaLD, lipid content of M. *longissimus dorsi*.

.

### QTL effects on carcass composition traits

The QTL analysis for carcass composition traits identified 13 genome-wide (p < 0.05 corresponding to F = 8.1) significant QTL in five genomic regions (Table 3). Additionally 55 chromosome-wise significant QTL (p < 0.05 corresponding to F > 3.6), which are considered as suggestive at the genome-wide level, were also identified (Additional file 4: Table S3).

**Table 3:** **Positions and effects of significant QTL for carcass and meat quality traits in the cross between GG and NZW rabbits**

**OCU/LG**	**Trait**	**Model** ^**1**^	**cM** ^**2**^ **(Mb)**	**Flanking markers** ^**3**^		**95% CI** ^**4**^	**F-value** ^**5**^	**a (SE)** ^**6**^	**d (SE)** ^**7**^	**VF**_**2**_ **%** ^**8**^
				**Left or direct**	**Right**	**(cM)**				
2	Hind part weight (g)	2	0.0 (29.01)	INRACCDDV0192		0.0- 18.0	10.10**	**5.82 (1.30)**	0.82 (1.86)	5.96
3	Bone weight fore part (g)	2	90.0 (131.74)	Sat3	INRACCDDV0203	28.5- 90.0	9.11*	**4.45 (1.39)**	**-6.33 (2.12)**	6.03
7	Kidney weight (g)	1	90.0 (155.45)	D7Utr4	D7L1B10	20.0 97.0	8.36*	**0.84 (0.21)**	0.19 (0.31)	4.97
7	Hot carcass weight (g)	1	91.0 (157.34)	D7L1B10	INRACCDDV0092	5.0- 98.0	11.02**	**64.83 (14.78)**	36.34 (22.10)	6.46
7	Reference carcass weight (g)	1	91.0 (157.34)	D7L1B10	INRACCDDV0092	5.0- 98.0	11.34**	**63.70 (14.45)**	38.50 (21.62)	6.64
7	Fore part weight (g)	1	91.0 (157.34)	D7L1B10	INRACCDDV0092	3.0- 98.0	8.69*	**23.86 (6.16)**	13.94 (9.21)	5.17
7	Intermediate part weight (g)	1	91.0 (157.34)	D7L1B10	INRACCDDV0092	62.0- 98.0	13.06**	**17.66 (3.76)**	**11.21 (5.62)**	7.57
7	Hind part weight (g)	1	92.0 (157.45)	D7L1B10	INRACCDDV0092	3.0- 98.0	9.95*	**21.58 (5.18)**	11.84 (7.68)	5.87
7	Meat weight fore part (g)	1	92.0 (157.45)	D7L1B10	INRACCDDV0092	4.0- 98.0	8.67*	**20.34 (5.42)**	13.08 (8.05)	5.75
7	Meat weight intermediate part (g)	1	92.0 (157.45)	D7L1B10	INRACCDDV0092	15.5- 98.0	11.49**	**13.91 (3.21)**	8.69 (4.78)	7.46
7	Meat weight hind part (g)	1	93.0 (158.19)	INRACCDDV0092	D7Utr5	3.0- 98.0	9.35*	**18.72 (4.62)**	8.92 (6.88)	6.16
9	Bone weight fore part (g)	1	61.0 (65.57)	INRACCDDV0010	INRACCDDV0146	35.0- 98.5	8.94*	**7.10 (1.74)**	-1.83 (2.50)	5.92
12	Drip loss (%)	1	94.0 (127.58)	INRACCDDV0201	INRACCDDV0176	0.0- 94.0	8.16*	**-0.29 (0.10)**	**-0.58 (0.19)**	4.87
19	Hind part weight (g)	2	45.0 (48.44)	INRACCDDV0071	INRACCDDV0193	28.5- 67.0	8.52*	**3.23 (1.36)**	**6.87 (2.18)**	5.07

^1^Model 1-standard QTL model with covariate birthweight; Model 2-standard QTL model with covariate reference carcass weight, ^2^Chromosomal location is given as pedigree-specific cM position; first marker on each chromosome was set at 0 cM. Estimated physical position between the flanking markers in Mb is given in parentheses; ^3^Flanking markers (left or direct and right) of the QTL peak; ^4^CI-confidence interval; ^5^F-value is F-statistic for QTL using standard one QTL model; ^6^a-additive effect; ^7^d-dominance effect; the direction of additive and dominance effects is given as GG-allele effect compared to NZW, bold values indicate significant effects if the estimate divided by the standard error > 1.96; ^8^phenotypic F_2_ variance (%) explained by the QTL; **highly significant at 1% genome-wide level (F-value ≥ 10.0), *significant at 5% genome-wide level (F-value ≥ 8.10); pH_45_ - pH value 45 min *post mortem*, pH_24_ - pH value 24 h *post mortem*, meat colour_45_ L*, a*, b* - meat colour traits lightness, redness, yellowness 45 min *post mortem*, meat colour_24_ L*, a*, b*- meat colour traits lightness, redness, yellowness 24 h *post mortem.*

**Figure 1:**
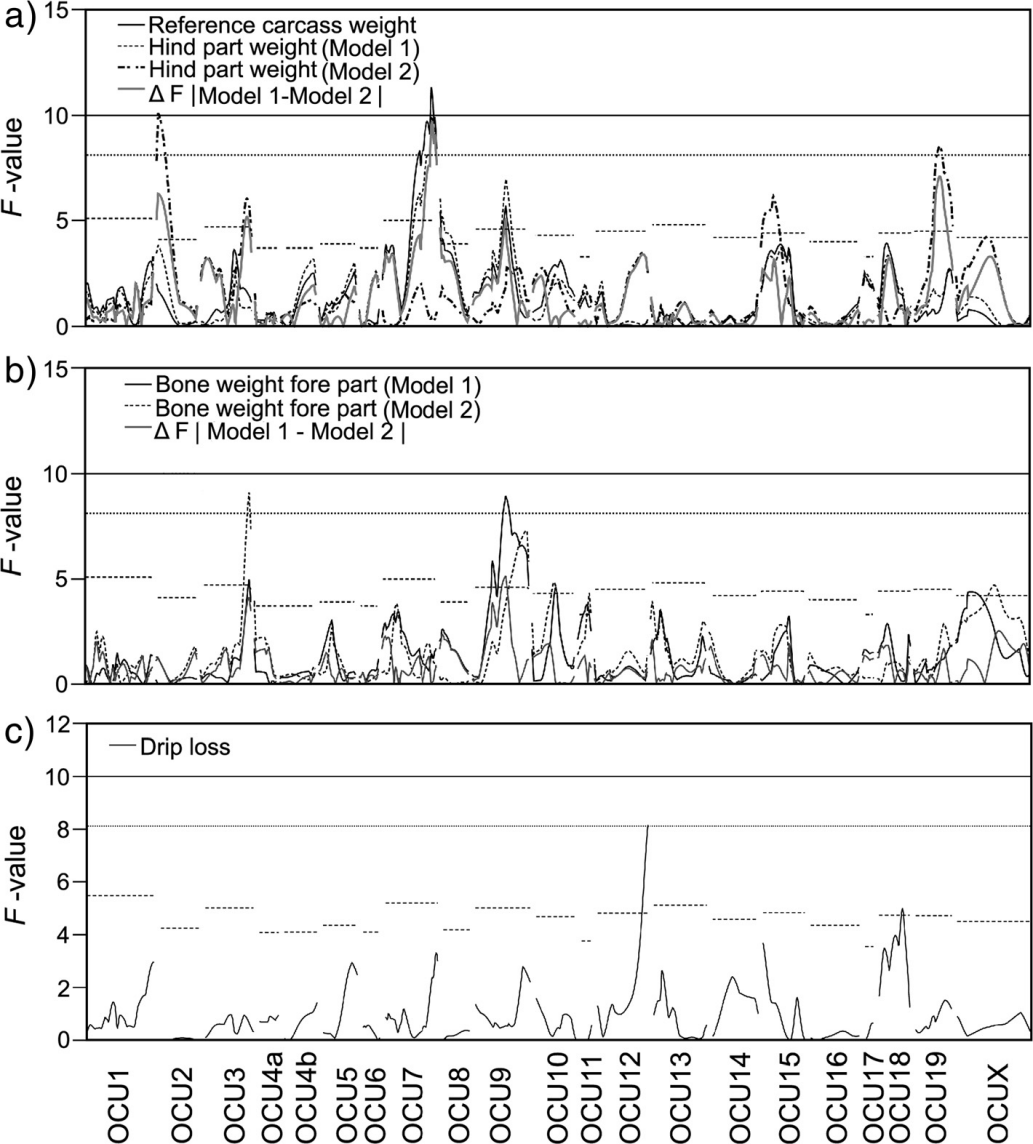
**F-value curves across all chromosomes for significant traits. (a)** Reference carcass weight, hind part weight with birthweight as a covariate (Model 1), and hind part weight with reference carcass weight as a covariate (Model 2), and for hind part weight ΔF = |Model 1 – Model 2| as the difference of F-values between the models 1 and 2. **(b)** Bone weights of the fore part with birthweight as a covariate (Model 1) and bone weights of the fore part with reference carcass weight as a covariate (Model 2), and for bone weights of the fore part ΔF = |Model 1 – Model 2| as the difference of F-values between the models 1 and 2. **(c)** Drip loss. The horizontal lines represent F-value thresholds at the genome-wide highly significant (solid), significant (dotted) and suggestive (dashed) levels of significance.

The most significant genomic region at the genome-wide highly significance level was mapped for carcass (F-value ≥ 11.02) and meat weights (F-value = 11.49) on OCU7 with a peak position between 91 and 92 cM (157 Mb, Figure 1). The QTL peak positions are located in the distal part of the q-arm of OCU7 within the flanking interval D7L1B10 (90.8 cM, 157.32 Mb) and INRACCDDV0092 (92.4 cM, 157.49 Mb). Consistent with the high phenotypic correlation between traits, this QTL was also highly significant for hot and reference carcass weights as well as for the total weight of the intermediate part and the meat weight of the intermediate part (F-value ≥ 11.02). These QTL accounted between 6.45 to 7.45% of the respective total F_2_ variance. In addition, the effect was significant for the carcass and meat weights of the fore and hind parts (8.67 ≤ F-value ≤ 9.95) and kidney weight (F-value = 8.36). This region has also a suggestive effect on liveweight.

**Figure 2:**
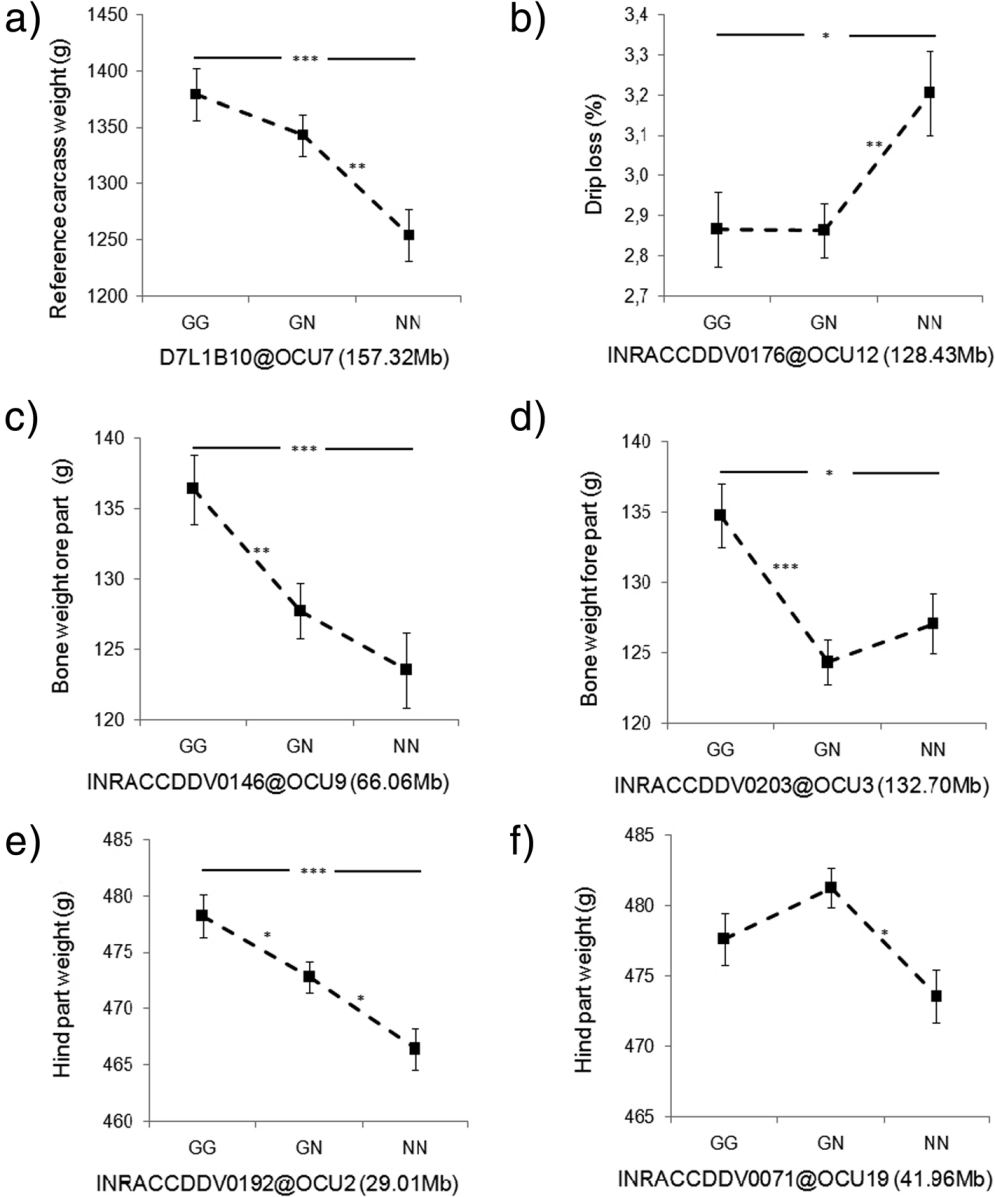
**Exemplary genotype effect plots of carcass traits at the nearest marker to the QTL peaks. a)** Reference carcass weight on OCU7 **b)** drip loss OCU12 c) and d) bone weight fore part on OCU9 and OCU3, respectively, **e)** and **f)** Hind part weight on OCU2 and OCU19, respectively, **a)**-**c)** using the model 1 with birthweight as covariate (Model1) **d)**-**f)** using the model 2 with reference carcass weight as covariate (Model 2), Values are LSM ± SE. G: Giant Grey allele, N: New Zealand White allele *P < 0.05, **P < 0.01 and ***P < 0.001 refer to significant differences between genotype classes (t-test).

Although the F-value curve for the different traits suggests the presence of a second QTL in the linkage group (Additional file 5: Figure S2), a two QTL model did not provide statistical evidence for the presence of a second QTL on OCU7. As expected from the differences between parental rabbit breeds the GG alleles increased all carcass and meat traits. The QTL effects were additive (Figure 2, Table 3, Additional file 4: Table S3). The identified major QTL on OCU7 is probably responsible for linear growth. Since the weights of all carcass parts and meat weights are affected by this QTL in the same direction and the correlation between these traits adjusted for the QTL on OCU7 genotypes is high (*r* > 0.9), pleiotropic effects on the development of carcass and skeletal muscles can be assumed. This assumption is further supported by the finding that the OCU7 QTL for the weights of the carcass parts and meat weight were lost when the reference carcass weight was included as a covariate into the model.

A genome-wide significant QTL for bone weight in the fore part (F-value = 8.94) was identified on OCU9 at 61 cM (65.57 Mb, Figure 1, Table 3). The nearest markers to the peak position of the QTL for bone weight in the fore part on OCU9 were INRACCDV0010/016 (60.2 cM, 64.72 Mb) and INRACCDDV0146 (61.5 cM, 66.06 Mb). QTL alleles of Giant Grey had additive effects on the bone weight in the fore part (Figure 2, Table 3). To the same region additional suggestive effects were mapped for bone weight in the hind part (F-value = 5.58), for the fore (F-value = 6.45) and hind (F-value = 6.96) part weights of the carcass, for liveweight (F-value = 7.34), hot carcass weight (F-value = 5.81), reference carcass weight (F-value = 5.72), and the head weight (F-value = 7.22, Additional file 4: Table S3). The QTL explained between 3.29 to 7.59% of the phenotypic F_2_ variance of the corresponding traits (Table 3, Additional file 4: Table S3). The QTL on OCU9 affected not only bone weights, but also carcass weights and fat content in M. *longissimus dorsi*. This QTL particularly influenced the fore and hind parts of the carcass including total mass, bone and meat weights. When reference carcass weight was included as a covariate in the one QTL model for bone weight in the fore part, the position of the highest peak of the bone weight in the fore part shifted from 61 cM (65.57 Mb) to 102 cM (113.67 Mb, Figure 1). The direction and magnitude of the additive effects of the two QTL were consistent (Table 3, Additional file 4: Table S3).

Using the reference carcass as covariate in the model (model 2), genome-wide QTL for hind part weight were observed on OCU2 at 0 cM (29.01 Mb) and OCU19 at 45 cM (41.96 Mb) (Table 3, Figure 1). The OCU2 QTL alleles of GG had additive effects and the OCU19 GG alleles were dominant (Figure 2, Table 3). These QTL explained 5.96% and 5.07% of the phenotypic F_2_ variance. With the model 2, an additional genome-wide significant QTL was identified for bone weight in the fore part on OCU3 at 90 cM (132.70 Mb, Table 3, Figure 1). QTL alleles of GG had overdominance effects (Figure 2, Table 3). The QTL accounted for 6.03% of the phenotypic F_2_ variance.

### QTL effects on meat quality traits

The QTL analysis for meat quality traits identified one genome-wide (p < 0.05) significant QTL on OCU12 (Table 3). Additionally 13 suggestive QTL at the chromosome-wise significance threshold of p < 0.05 were identified on chromosomes 1, 2, 5, 8, 9, 11, 16, 17 and 18 (Additional file 4: Table S3). The genome-wide scan for meat quality traits identified a significant QTL on OCU12 affecting drip loss of the whole carcass (F-value = 8.16, Figure 1). The peak QTL position is located at the end of the q-arm at 94 cM (127.58 Mb) near the marker INRACCDDV0176. The QTL accounted for 4.78% of the phenotypic F_2_ variance. The GG QTL allele had negative dominance effects (Figure 2, Table 3). Another QTL for drip loss which was suggestive was mapped on OCU18 (F-value = 5.00, Figure 1, Additional file 4: Table S3).

Since 68 QTL for carcass composition and meat quality traits are suggestive further studies are needed to confirm their effects. Therefore, these QTL are listed in Additional file 4: Table S3, but are not further discussed here.

### Candidate gene identification

Previously, a single marker association analyses of the *MSTN* gene, as a key candidate gene affecting muscle development in different species [30-32], identified association of myostatin variants with several carcass composition traits in rabbits [11]. In our candidate gene study, out of three SNPs in the *MSTN* gene, only SNP c.373 + 234G > A [GenBank: NM_001109821] showed a significant association, while the SNPs c.-125T > C and c.747 + 34C > T were not significant. The F-value curve pertaining to the linkage analysis across the whole chromosome 7, suggests the presence of a major peak for different carcass traits at the end of the chromosome in addition to the QTL effects on the same traits 6 cM away from the *MSTN* gene (Additional file 5: Figure S2). However, the two QTL analysis in the examined F_2_ population did not reach the significance level to provide evidence for the existence of a second QTL different than that identified by the single-QTL analysis at position 90–93 cM.

The estimated confidence intervals for all identified QTL effects were very large and cover almost the whole chromosome. Therefore, the selection of putative candidate genes is difficult and requires further studies to reduce the confidence intervals. Considering the confidence interval (62.0 to 98.0 cM) of the main QTL peak on OCU7, the search in the rabbit genome database (http://www.ensembl.org/Oryctolagus_cuniculus/, Ensembl release 73, OryCun 2.0) provides a list of about 300 genes. For example, the insulin like growth factor binding protein 2 (*IGFBP2,* 7:158.054321Mb) and the insulin like growth factor binding protein 5 (*IGFBP5*, 7:158.093549Mb) are located near directly under the peak position of the OCU7 at 158 Mb. These genes are positional and functional candidate genes for effect on carcass weights. *IGFBP2* gene effects associated with growth and carcass composition were reported for chicken [33] and pigs [34]. In addition, an overexpression of *IGFBP2* reduces the postnatal body weight gain in transgenic mice [35]. A common QTL region between sheep on OAR2 and cattle on BTA2, which is orthologous to the rabbit OCU7 QTL region, have been previously reported for carcass weight, eye muscle area and retail product yield [36].

## Conclusions

This study provides a comprehensive genetic map of 189 markers for the rabbit genome. The marker linkage map as well as the link to the physical map provides valuable information for the further improvement of the rabbit genomic sequence assembly and a tool for mapping functional effects. In addition, this study was the first QTL analysis in rabbits for carcass composition and meat quality traits. The major QTL on OCU7 for carcass and meat weights, the QTL for bone and carcass weights on OCU9, as well as the QTL for drip loss on OCU12 have not been described before. This genetic information provides an important step in the identification of functional quantitative trait genes. Fine mapping in an advanced intercross population and in particular association mapping in breeding populations using dense SNP markers will facilitate candidate genes identification in the future.

## Methods

### Animals

For linkage analyses an F_2_ intercross population with 363 offspring (183 males and 180 females from 9 F_1_ bucks and 33 F_1_ does) was generated from an initial cross between six purebred GG bucks and six purebred NZW does (Additional file 6: Table S4). GG and NZW rabbits were obtained from local breeders. Rabbits were housed under standardized conditions in the experimental station of the Department of Genetics and Animal Breeding of the Agricultural University of Krakow. Adult rabbits were housed in two-storey wooden cages which were placed in a heated hall with lighting and exhaust ventilation. Cages were equipped with a water supply system (nipple drinkers). Offspring were weaned at the age of five weeks and subsequently housed in metal cages arranged in batteries with two rabbits per cage. Rabbits had *ad libitum* access to feed and water. The feed consisted of 16.5% protein, 14% crude fibre, and 10.2 MJ metabolisable energy*.* The experiment was approved by the Agricultural University of Krakow.

### Phenotypes - carcass composition

The animals were slaughtered at the age of 12 weeks. After removing the skin, the head and the giblets, the weights of liver, kidney, lung, heart, head and hot carcass (without head and giblets) were recorded. Afterwards, the carcass was first kept at room temperature in a ventilated area for 45 min and then at 4°C until 24 h *post mortem*. After cooling, the carcass was weighted to determine the reference carcass weight. Then the carcass was cut to the fore (cut after the last rib), intermediate (cut after the last lumbar vertebra) and hind part and further dissected to meat, bone and dissectible fat. All carcass parts were weighted. The scapular, perirenal and inguinal fat percentages were calculated as percentage of the appropriated carcass part.

### Phenotypes - meat quality

The pH values in the M. *biceps femoris* were measured at 45 min and 24 h *post mortem* using a pH meter with an accuracy of 0.01 (HI-9024). Meat colour was measured on the surface of M. b*iceps femoris* according to the CIELab standards (CIE 1976: light source D65 and 8 mm diameter) at room temperature (20°C) at 45 min and 24 h *post mortem* with a CR-400 Minolta chromometer (Minolta Co., Ltd., Osaka, Japan). The values of lightness (L*), redness (a*) and yellowness (b*) were recorded. The shear force by Warner-Bratzler was determined in a fresh M. *longissimus dorsi* sample (14mm diameter, 15mm high) with the Texture Analyser TA-XT2 (Stable Micro System, Goldaming, UK) using a triangular knife incision. Drip loss was calculated as percentage of the weight difference between hot and reference carcass weight to the hot carcass weight. Protein and lipid content in M. *longissimus dorsi* were determined according to ISO standards. The protein content was determined by the method of Kjeldahl (PNA-04018:1975). The lipid content was determined using the method of Soxhlet (PN-ISO-1444:2000). Some phenotypes could not be measured in purebred animals of GG and NZW. These are the traits for meat colour in NZW and for shear force, protein and fat content in GG rabbits.

### Genotyping

For genotyping, the DNA was extracted from 200 μL whole EDTA blood using NucleoSpin® Blood kit (Macherey & Nagel, Düren, Germany). Microsatellites were amplified by locus-specific PCR and fragment size was determined by the LI-COR DNA Analyzer 4200 (LI-COR Biosciences, Lincoln, USA) as described in detail, previously [37]. Initially, we tested 387 available rabbit microsatellites [2-10] with all parental animals to identity informative markers for the cross between GG and NZW. Nine markers were fully and 180 partially informative. These markers were genotyped in all F_2_ animals. 122 markers were uniform and 76 markers could not be successfully amplified or did not give specific fragments (Additional file 1: Table S1). The polymorphism information content in the F_2_ population was calculated according to Botstein [38].

Since numerous mutations in the *MSTN* gene had been associated with growth, muscle mass, and other carcass composition traits in different species [30-32,39-43], the *MSTN* gene was chosen as a functional candidate gene. We additionally genotyped three SNP (c.-125T > C, c.373 + 234G > A, c.747 + 34C > T) in the rabbit *MSTN* gene [11] by allele specific PCR [44]. To detect genotyping errors, the observed F_2_ genotype frequencies were compared with the expected frequencies using a chi-square test. This analysis revealed three microsatellite markers (INRACCDDV0035, INRACCDDV0157 and INRACCDDV0204) with null alleles, which were excluded from further analyses. Furthermore, we checked recombination frequencies and double recombinations between adjacent markers to detect potential genotyping errors. For checking recombination events and counting the number of informative meioses per locus we used the CHROMPIC and PREPARE options, respectively, from the software package CRI-MAP, version 2.4 [45].

### Construction of a pedigree specific linkage map

The pedigree specific linkage map for the studied population was built on the basis of 186 microsatellite markers and three SNP markers using Kosambi mapping function in CRI-MAP software, version 2.4 [45]. In the first step, a two-point linkage analysis was performed in which all markers were analyzed against each other. In the second step, the marker locus order was calculated with the BUILD option allowing different recombination rates in the intervals between the sexes. The BUILD option was started with the highest informative loci. Subsequently, the other loci were consecutively included for the construction of linkage groups. The FLIPS option was used to confirm the correct order of the marker loci. Finally, we generated sex-specific and sex-averaged genetic maps. The genetic distances are given in centiMorgan (cM) between markers, with the first marker of every linkage group at 0 cM. The physical positions of markers in megabase (Mb) were given according to the respective sequence position in the rabbit genome assembly at ENSEMBL (http://www.ensembl.org/Oryctolagus_cuniculus/, Ensembl 73, OryCun 2.0). Peak QTL positions were translated into physical positions as a linear genetic distance between adjacent markers with physical positions.

### Basic statistical analysis

Basic statistics were performed using the PASW software package version 18.0 (SPSS, Inc., Somers, NY, USA). The phenotypic data were checked for normal distribution using the procedure EXAMINE (Kolmogorov-Smirnov test). Pearson’s correlation coefficients between traits were estimated using the CORRELATE procedure. Family (full-sibs), sex and season were detected as factors affecting the phenotypes using the GLM procedure and thus were considered as fixed effects in the QTL model. Genotype effect plots were drawn with least square means (LSM). A t-test with Bonferroni correction for multiple testing was performed to test phenotypic differences between genotype classes of the nearest marker to a QTL peak.

Single marker analyses were performed for the three *MSTN* SNPs. The model included common litter effects, season, sex, SNP genotype, interaction between season and family as fixed effects, and birth weight as covariate (PASW, Version 18.0). For pairwise comparisons, p-values were adjusted for multiple testing using the Bonferroni procedure [11].

### QTL mapping

QTL mapping was performed on the basis of the sex-averaged map using Grid-QTL [46]. The BC-F_2_ module was used which assumes that founder lines are fixed for alternative alleles at QTL loci. Data were analysed with least squares regression interval mapping method using family (full-sibs, 36 levels), sex (2 levels) and season (4 levels) as fixed effects, and birth weight as an interactive covariate (model 1). Reference carcass weight was highly correlated with the weights of the carcass parts as well as the individual meat, bone and fat weights of the carcass parts (0.23 < *r* < 0.99, p < 0.001). Therefore, it was included as a covariate in additional analyses (model 2). Genome-wide and chromosome-wise significance thresholds were determined by permutation tests [47]. One thousand permutations were performed for all traits. Threshold values for a given level of significance were calculated as an average of thresholds over all traits. The F-value of 10.0 corresponds to genome-wide highly significance (α = 0.01) and the F-value of 8.1 to genome-wide significance (α = 0.05). Genome-wide suggestive QTL are detected at chromosome-wise significance of α < 0.05, corresponding to F-value thresholds between 3.6 and 6.0 for the different chromosomes. The 95% confidence interval of a QTL was estimated using parametric bootstrap analysis with 1000 iterations [48]. OCUX was analysed as a pseudo-autosome in all analyses as all markers were located in that region. The direction of the genetic effects was given as GG allele effect compared with NZW. QTL positions are given as cM distance of the highest F-value from the first marker on a chromosome. The phenotypic variance explained by a QTL was calculated as reduction of residual sum of squares in the full model (with QTL) compared with the reduced model (without QTL).

A 1 cM grid search was performed in Grid QTL by fitting model to estimate the effects of two QTL at separate positions within the same linkage group simultaneously, examining all possible pairs of markers, to test whether the two-QTL model explained significantly more variation than the best QTL from the one-QTL analysis. Two F-statistics were computed. The two-QTL model was accepted if there was a significant improvement over the best possible one-QTL model at p < 0.05 using a variance ratio (F) test.

## Additional files

Additional file 1: Table S1. Information about tested markers. Additional file 2: Table S2. Summary of the information for markers that were integrated into the genetic map [1-4,6-8,10,12-17]. Additional file 3: Figure S1. Cytogenetic associated linkage map for the F_2_ cross GG x NZW. The cytogenetic map (left) of every chromosome is connected to the sex averaged pedigree-specific linkage map (right). The numbers on the right hand side of the linkage maps give the estimated distances between loci in cM (Kosambi), the statistical support for the pair-wise order of markers is given on the left hand side. The total genetic length of every linkage group is given at the bottom of each bar. Connecting lines indicate the cytogenetic positions of microsatellites previously mapped by fluorescence in situ hybridization [1-4,7,12-15,17]. Additional file 4: Table S3. Positions and effects of suggestive QTL for carcass traits of the cross between GG and NZW rabbits. Additional file 5: Figure S2. F value curves across OCU7 pertaining to QTL scans for carcass traits (Model 1). Additional file 6: Table S4. Structure of the F_2_ pedigree (GG x NZW).

## Abbreviations

GG: Giant grey
NZW: New Zealand White
BAC: Bacterial artificial chromosomes
GWAS: Genome-wide association study
LD: Linkage disequilibrium
MSTN: Myostatin
OCU: Oryctolagus cuniculus chromosome
SNP: Single nucleotide polymorphism
Bp: Base pairs
Mb: Mega base pairs
QTL: Quantitative trait locus
