# Supplementary material for: A comprehensive linkage map and QTL map for carcass traits in a cross between Giant Grey and New Zealand White rabbits
Source: BMC Genet. 2015 Feb 11;16:16. doi: 10.1186/s12863-015-0168-1 (PMC4330979; doi:10.1186/s12863-015-0168-1)
Supplement: Additional file 1: Table S1. — Information about tested markers. [file 12863_2015_168_MOESM1_ESM.docx]

# Additional file 1

## Table S1: Information about tested markers

| Microsatellit (*Associated gene*) | Accession number | Primer forward | Primer reverse | PCR result | Microsatellite Reference |
| --- | --- | --- | --- | --- | --- |
| ACE2 (*DCP1*) | M58579 | TCCTGAGACATCCAGGCCGG | ATGGTCAGTGGCCTGGGCTG | not polymorph | Korstanje et al. 2001a |
| CCCCG *(HBA)* | M35026 | TGGGCTCCGCACACTTCTGG | GACAGGGCGCCGGGCAGGTCGTCC | no fragment | Ferrand et al. 2000 |
| D0Utr1 *(CACNLB1)* | AF389356 | TCCACGCAACCATTTCTGGA | CCAGCGGGTTTGGAAAATCA | not polymorph | Korstanje et al. 2001a |
| D0Utr2 *(CACNLB1)* | AF389360 | TAAGTTAAGAATAGTATCAAATCA | ATGGGGAGTAGAGCAGCAGA | no fragment | Korstanje et al. 2001a |
| D0Utr3 *(CAPN4)* | M13364 | AGTCGCTGCATGAGTCACGG | CTGATGACTCCGCCCAGGAT | no fragment | Korstanje et al. 2001a |
| D0Utr7 *(MYH7)* | AF192306 | GAGTGAATCAATGGGCGGAAGACC | ACTGGGCAAATGGAAAATCAGC | no fragment | Korstanje et al. 2001a |
| D0Utr8 *(MT2)* | X07791 | AGGTGCTTTTCCTGGCCTCC | GCCCATATGAGATGCCAGCC | not polymorph | Korstanje et al. 2001a |
| D0Utr10 *(CYP2C4)* | M74203 | TCCCTGAAAATGGCAACCATAT | TCAAGGAGAGCAGGGAAATTATTG | polymorph | Van Haeringen et al. 1996 |
| D0Utr16/D12L1C2 | AF421939 | AGGGGCCTCCATCCTCTACA | ATTATGTGTCAGGCAGGCTGTGTC | polymorph | Korstanje et al. 2003 |
| D1Utr1 | AF389372 | TGTATGCCATGCTTTTAGTATTA | TTTGCCCATTCTTTTGGTAT | no fragment | Korstanje et al. 2001b |
| D1Utr2 | AF389367 | CTGGGTAACTTTATATTTTCACAA | CACCCACCCCTACTCAAC | polymorph | Korstanje et al. 2001b |
| D1Utr3 | AF389359 | AAGACCCTGGTGCATGGC | AACACTTTTGGGAACAAAGAAAGT | polymorph | Korstanje et al. 2001b |
| D1Utr4 | AF389353 | TAACTGGTGGGCCAAATGCC | TGCCACCCATGTGGGAAAG | polymorph | Korstanje et al. 2001b |
| D1Utr5 | AF389357 | TTTGTTGTGGGCATTTGG | GGATCATATTGTCCTGTTAGCC | polymorph | Korstanje et al. 2001b |
| D1Utr6 | AF389354 | GACCCTAGTTGTCCCAACACA | ATTGCAGCCAACTGGGGA | polymorph | Korstanje et al. 2001b |
| D1Utr7 | AF389355 | GGAACCTGCTTAGTCTCCTGCA | GCAGGCATGCAAGAAAAACAG | polymorph | Korstanje et al. 2001b |
| D1L1B10 | AF398352 | GACAACATCTGCTAATGCTTG | TCTCTGCAGGCTCATTCTC | polymorph | Korstanje et al. 2001b |
| D1L1G3 | AF389356 | TGGAGGGGGAAGAAAGAAA | TGACTTCAGCCTGGGCC | no fragment | Korstanje et al. 2001b |
| D1L2B4 | AF389358 | TGGGGATGCAGGTGTCTCA | GGTTTGCCTAGATTTCAAGGCT | polymorph | Korstanje et al. 2001b |
| D1L5B11 | AF389362 | GGAGCCAACGTAGGAAAATCAAGT | TCAAGCCAAGTATAGAGCAGAAAA | not polymorph | Korstanje et al. 2001b |
| D1L5G7 | AF389362 | GGCCTCATATCACGTAACATCC | GCCATCTTGCTTCCCTGAGT | no fragment | Korstanje et al. 2001b |
| D1L5H4 | AF389364 | GAAGCCATTGTAATCCATAAA | GCTGTAAACGAAAAGGTA | no fragment | Korstanje et al. 2001b |
| D1L6B2 | AF389365 | ATACGGGACTGCCATCT | TCTTCTATTCTTTACCTTGTCTAC | no fragment | Korstanje et al. 2001b |
| D1L6C1 | AF389366 | AGGATTTGGCAGTATTTTTATTG | CTTTAGCCCTTCCCAGATGA | not polymorph | Korstanje et al. 2001b |
| D1L7B12 | AF389368 | GGGGGAAAGTCATTGTAA | GCAGGCATCAGGCATTTG | no fragment | Korstanje et al. 2001b |
| D1L7C11 | AF389369 | ATGGCACCAATAATGTC | TTTGGCAATAGATGAGG | polymorph | Korstanje et al. 2001b |
| D1L8A11 | AF389371 | CCAGGAAGTGAATCAGTGGATAA | GCGGGCCCGAGGAATGA | no fragment | Korstanje et al. 2001b |
| D1L8A7 | AF389370 | AATGCTGGCTTGAAGTCCTAAA | CATGTGAGTGAGTTCTGTATTGTA | no fragment | Korstanje et al. 2001b |
| D1L8B6 | AF389373 | GAGACTTGGATGCGTTACA | GATCCCGAGAGTGAAGAAGG | no fragment | Korstanje et al. 2001b |
| D1L8C9 | AF389374 | TTAGTATGGGGGAGAGTTGA | ACCTTGTAGCTTAATCCTGTT | polymorph | Korstanje et al. 2001b |
| D1L8D5 | AF389375 | TTAATGGGAGGTAATCTC | TAACCCCTGAATCTAATA | no fragment | Korstanje et al. 2001b |
| D3Utr1 *(FABP6)* | Z54345 | TGCCCCACAGTTCTCA | CAAGGGCTGGTGGTAG | no fragment | Korstanje et al. 2001a, 2003 |
| D3Utr2 | AF421903 | AGGAAGTGAGGGGAGGTGTT | ATAATGTGCTGCCAAAATAGAAAT | non specific fragment | Korstanje et al. 2003 |
| D3Utr3 | AF421904 | ATTTGCAGTTTTATTCAT | AACTGCCCTTGTCTTTC | no fragment | Korstanje et al. 2003 |
| D5Utr1 *(MT1)* | X07790 | CCAGCCGTTGCAGCCAGTTG | ACTCCAGGATGCCCCCAGGG | non-specific fragment | Korstanje et al. 2001a; 2003 |
| D5Utr2 | AF421913 | TCTCCTCCCTGATTTTCTCTGTAT | GTTAAACTCAAATGGGTGCTCTC | polymorph | Korstanje et al. 2003 |
| D5Utr3 | AF421905 | TGGCTTTGCAGGCAGTGGTTT | CTCTTTCTTTCTCTTTTCCTCTC | not polymorph | Korstanje et al. 2003 |
| D5Utr4 | AF421907 | AAAGTGAGCCTGCAGATGAGAGCA | GGGCGGGGCGGTTACAGT | polymorph | Korstanje et al. 2003 |
| D5L1A11 | AF421906 | TGGGAGAAGGAAACAAA | GCCATCACGTCTTAGGA | no fragment | Korstanje et al. 2003 |
| D5L1C11 | AF421910 | GCTGCTTTGGCTCCTAATGTGT | CTTACCGGGAAATCTCTGACCT | no fragment | Korstanje et al. 2003 |
| D5L1C3 | AF421908 | CAGCGGTAAGAGTGAGAAAC | TCCCCCATAACAAAAGAGG | polymorph | Korstanje et al. 2003 |
| D5L1C9 | AF421909 | GCTCTTGGCTCCTGGTTTC | AGAGTTCTCCGTCCCTGATGG | not polymorph | Korstanje et al. 2003 |
| D5L1D7 | AF421911 | AGGTGGGTGAGGAGACC | TTGTAATCGGCTCACTAT | no fragment | Korstanje et al. 2003 |
| D5L1E8 | AF421912 | CCAGCTGGTAATAGTAGAGA | AAGGCATTTGTGGAGTGAA | no fragment | Korstanje et al. 2003 |
| D6Utr1 *(HBA)* | M74142 | AGGGGCTGCTGAAAGAACCC | CTGCACTCGACCTCCGGTTT | not polymorph | Korstanje et al. 2001a; 2003 |
| D6Utr2 | AF421922 | CGAGCTCCTTGCTGCATGAC | AGGGCGACCAGCGGTCTAT | polymorph | Korstanje et al. 2003 |
| D6Utr3 | AF421915 | CCTGGGGGTTTCTTTTGTGTC | GTTAGCAGCGTGTACCCATTTC | not polymorph | Korstanje et al. 2003 |
| D6Utr4 | AF421916 | CAGAAGGGCATTTGTTTTG | GGTGATTCTTTCTTCTGCCTCTTA | polymorph | Korstanje et al. 2003 |
| D6Utr5 | AF421923 | AAGACTTATTTATTTATTTG | AGGAACTTGGCTGGAT | no fragment | Korstanje et al. 2003 |
| D6L1D7 | AF421914 | GCCTGTTCTGCCTTTTTCTG | AACACCTTCCTCTGGCTTCTG | non-specific fragment | Korstanje et al. 2003 |
| D6L1G8 | AF421917 | CCTGTAAATCCTTGGCTGCTGGTA | CGGCTTATGTGGGCTGCTTCC | non-specific fragment | Korstanje et al. 2003 |
| D6L2B5 | AF421918 | AACAGCCCCAGATTTTG | AAGTGCAGGTTGTATTTTGAT | polymorph | Korstanje et al. 2003 |
| D6L2F1 | AF421919 | TATCACCCTTGCTGCCTTCT | ACCCTCAAAATAATAATCCAACTG | polymorph | Korstanje et al. 2003 |
| D6L2H3 | AF421920 | CAAATGAAATGAGGAGGGAGATA | CATTGAGGACATTTGAGTAGTGAG | polymorph | Korstanje et al. 2003 |
| D6L3A4 | AF421921 | TTGGGGGTGGTGGTGGTGA | CCCCTTCGCCCCGTAACTG | not polymorph | Korstanje et al. 2003 |
| D6L3F8 | AF421924 | CTCCTGCCCTGTTCTAT | CAGGCTGGTCTTATTAC | no fragment | Korstanje et al. 2003 |
| D6L3H10 | AF421925 | GGCTCAGATGACCCTATTCC | GGCTTTCCTTCTTCTTCTTCAG | polymorph | Korstanje et al. 2003 |
| D7Utr1 *(TCRB)* | M26312 | AGCCATTCCTACCGCAACCA | TGCACGTGTGTGTGCATGTG | not polymorph | Korstanje et al. 2001a; 2003 |
| D7Utr2 | AF421934 | GATTATTTATAGCCCTTCTCCA | TCTTCCCGTTCATTGTGTT | no fragment | Korstanje et al. 2003 |
| D7Utr3 | AF421935 | TAGTGAGCCTCTAACTTCTGTA | CCCTAACATGGGGAAATG | polymorph | Korstanje et al. 2003 |
| D7Utr4 | AF421932 | TGCTAATGTGCCCAGAAAGGTA | GGCATCCCAAAAGGCAGTAT | polymorph | Korstanje et al. 2003 |
| D7UTR5 | AF421930 | ACACCTGGGGAATAAACAACAAG | GAGGGAGGCAGAGGGATAAGA | polymorph | Korstanje et al. 2003 |
| D7Utr6 *(PODXL)* | NM_001082766 | GTGGCCACGTGTTAATCTATCTTG | ACTCCAGCCAGGGTCACATTTTA | polymorph | Korstanje et al. 2003 |
| D7L1B10 | AF421926 | TTGGCAGGAAGAAAAGGAAGATT | TTTGTCATAAGCATTTGGGAAGTG | polymorph | Korstanje et al. 2003 |
| D7L1C4 | AF421927 | CTGGCCCAGGACTGACCT | CCCCAGATAACATTTCTATGGA | not polymorph | Korstanje et al. 2003 |
| D7L1E2 | AF421929 | GCAGCCATCTTGCAACCATAA | TTTCAGCCTGGCCCACTATTT | not polymorph | Korstanje et al. 2003 |
| D7L1H10 | AF421931 | GCTGCTGCATCTCAACTG | TACATGGGAAGGATTACTGG | not polymorph | Korstanje et al. 2003 |
| D7L2F2 | AF421933 | TAGGCATTTAGGGAGTGAAC | GGAGGGGGATGGTAGAG | polymorph | Korstanje et al. 2003 |
| D10Utr1 *(WAP)* | NM_001082390 | TTGCTTACAGAGTCCAGAAAACC | CTTGTACCCACGGCTCAGAG | polymorph | Korstanje et al. 2001a |
| D12Utr1 *(RLADPA1)* | M22640 | GCCAGACCCCAAAACACAAA | TCATGGGCTCAATGCTTGTC | polymorph | Korstanje et al. 2001a; 2003 |
| D12Utr2 | AY095444 | CAGGGTGCACCTCAACT | TCCTGTAGATGGATGAAAGA | not polymorph | Korstanje et al. 2003 |
| D12L1A4 | AF421937 | TACGGAAAGGTATCTGTACTTC | AAACATGAATTTGTCTCCCTTATA | no fragment | Korstanje et al. 2003 |
| D12L1B2 | AF421938 | GGGTACAGATAACACTTTCA | GGTATCCTAATCCACTTTG | not polymorph | Korstanje et al. 2003 |
| D12L1C2 | AF421939 | AGGGGCCTCCATCCTCTACA | ATTATGTGTCAGGCAGGCTGTGTC | no fragment | Korstanje et al. 2003 |
| D12L1D8 | AF421940 | CCAAAGCAATTAGAAAAGAAAA | TTGCACGTAAAGAGAAATGG | not polymorph | Korstanje et al. 2003 |
| D12L1E11 | AF421941 | AGTGGTAGCGCTTTGGTCTG | GCTCCTTGGGGCATTTG | polymorph | Korstanje et al. 2003 |
| D12L1H3 | AF421943 | GGGCTTTAACCCTCTGTGTCA | AAACCAGTGAGGGTGGCAAAGAC | polymorph | Korstanje et al. 2003 |
| D12L3G10 | AF421944 | AGATGGTGCCTCTAAAAACTGAA | GGGAAGCCATTGTATCCATAAG | not polymorph | Korstanje et al. 2003 |
| D12L4A1 | AF421945 | GCTAATTACCCAAAGGAACATACA | CAGTGCAAATTTGGAAGGTCT | polymorph | Korstanje et al. 2003 |
| D12L4E10 | AF421946 | CCGGCAGTGAGGTAAAGA | GGGTGGTGGTAAGGTT | no fragment | Korstanje et al. 2003 |
| D12L5A6 | AF421947 | GGTGTGAACCACTAGATAGAA | CAAAATTAGGTCCCTTGTAGT | not polymorph | Korstanje et al. 2003 |
| D19Utr1 *(ALOX15)* | M33291 | AGAGACCACCCCAGAATCCTCT | ATGGAGCTCCTGGCTTGTTG | not polymorph | Van Haeringen et al. 1996 |
| D19Utr2 | AF421952 | AGTTGCTCCCACCCGATTTTA | TGCTGTTGGGAGTAGATTGACC | not polymorph | Korstanje et al. 2003 |
| D19Utr3 | AF421951 | AAATTCATGTTCCCTTCTCT | ATTCATGGCATCACTTTG | polymorph | Korstanje et al. 2003 |
| D19Utr4 | AF421949 | CGACCGTGGGCTCAGAAGAA | TGTATGTGGGTGTGGGTGTAGAG | polymorph | Korstanje et al. 2003 |
| D19L1A4 | AF421948 | AGGCACCGGGTTCTTGAGCAG | AGCAGGGCCAGCCACACTTGAT | no fragment | Korstanje et al. 2003 |
| D19L1E12 | AF421950 | CATGGGCTGAGTGGATAGGAA | TACTGTTGCTTGCTGGGATTTTTA | polymorph | Korstanje et al. 2003 |
| DXUtr1 | AF389361 | TTTGTTGAAGAGGCTGTGCTATT | CTTGTGGCCATTTGGAGAGTGAAC | polymorph | Korstanje et al. 2001b |
| INRACCDDV0001 *(CALR)* | AJ874368 | CATTCGCTGTCTCAATCCAA | ACATGGGTACATGCCAACTG | no fragment | Chantry-Darmon et al. 2005 |
| INRACCDDV0003 *(PIGR)* | AJ874369 | GATCAGCGAGCGCCTCTC | TCCATCTGAATGAGGCACAA | not polymorph | Chantry-Darmon et al. 2005 |
| INRACCDDV0004 *(CALR)* | AJ874370 | ACACAATTTGCTTCGTGCAG | TTCCGATTTGCAGGATTTTT | polymorph | Chantry-Darmon et al. 2005 |
| INRACCDDV0005 *(GPX1)* | AJ874371 | GTGAACCAACGGAAAAGGAA | TTCCACTGCCCACAATTCTT | polymorph | Chantry-Darmon et al. 2005 |
| INRACCDDV0006 *(ICAM5)* | AJ874372 | CACCCTTGCACAGACAGAAA | ACCAACGGCAAAGGAAGAC | polymorph | Chantry-Darmon et al. 2005 |
| INRACCDDV0007 *(MT1A)* | AJ874373 | CTGCTAGCTCTGGGTGGAAG | TGTGTGACCTTGTGGCCTTA | not polymorph | Chantry-Darmon et al. 2005 |
| INRACCDDV0009 *(DNASE1)* | AJ874374 | CAGGAATACTTGCAGCACA | ACTCAGTGCCTCGATTAGGG | not polymorph | Chantry-Darmon et al. 2005 |
| INRACCDDV0010 *(NPC1)* | AJ874375 | GAACCAGCAAATGGAAACTCA | CCAAGGTCTGAAAGTCATTGC | polymorph | Chantry-Darmon et al. 2005 |
| INRACCDDV0012 *(MT1A)* | AJ874376 | GGAACAGCCAGGTCTTGAAC | GTGAACCAGTGAGTGGCAGA | not polymorph | Chantry-Darmon et al. 2005 |
| INRACCDDV0014 *(SLC2A1)* | AJ874378 | ATAAGCCTTCCCAAATTGCAG | TGAGCAGCTCTGGTAGAGGTT | polymorph | Chantry-Darmon et al. 2005 |
| INRACCDDV0016 *(NPC1)* | AJ874380 | TATTCCCCCAACTCTGTCCA | CAAGCGTGTAAGTGCCAAGA | polymorph | Chantry-Darmon et al. 2005 |
| INRACCDDV0017 *(CYB5)* | AJ874381 | GGCATTTGGGTGAGTAAACC | CACGTGGAGAGCAACATCTG | polymorph | Chantry-Darmon et al. 2005 |
| INRACCDDV0018 *(CSN1S1)* | AJ874382 | GGGGAGTAAACCAGCAGATG | CCTCATTCGATTCATCTTCATAA | polymorph | Chantry-Darmon et al. 2005 |
| INRACCDDV0021 *(SLC15A1)* | AJ874384 | GGTCAACAGCCCTGTAGCAT | TTGAGGTGAGCCAGTGAATG | polymorph | Chantry-Darmon et al. 2005 |
| INRACCDDV0022 *(ERBB3)* | AJ874385 | GTTTAGCAAGTGGGGGAAGG | GGAGATGGAGCTGCACTGTT | polymorph | Chantry-Darmon et al. 2005 |
| INRACCDDV0023 *(CYP2C18)* | AJ874386 | GGTGGCAGGGACACACTAAT | GAACCAGCGAAATGGAAGAC | polymorph | Chantry-Darmon et al. 2005 |
| INRACCDDV0025 *(STK17A)* | AJ874388 | CATGGAGGACAAGACCACCT | AAGGAGTTTTCTGCGATAGTGC | polymorph | Chantry-Darmon et al. 2005 |
| INRACCDDV0027 *(CD1B)* | AJ874390 | TCCACAAAGAGAGAACACAGG | GCCAGACAATTGCTGTTCAA | polymorph | Chantry-Darmon et al. 2005 |
| INRACCDDV0029 *(CYP2C18)* | AJ874392 | ATCGTTTTCCCACTGACTGC | TGATAGCTGAAAGCCTCGTG | polymorph | Chantry-Darmon et al. 2005 |
| INRACCDDV0030 *(CA12)* | AJ874393 | GCAGTAGAAGATGGCCCAAG | TGAAAGGCAGAGTTACAGAGAGG | non-specific fragment | Chantry-Darmon et al. 2005 |
| INRACCDDV0031 *(CA12)* | AJ874394 | GCTCCAGATTCATGCAGTCA | TCTCAGTTTGTAACGTACTTGCTGT | not polymorph | Chantry-Darmon et al. 2005 |
| INRACCDDV0032 *(CSN3)* | AJ874395 | TTTTCCAGTTCTGCCATTCA | CCATTTGGTTGGAATATAAGGA | not polymorph | Chantry-Darmon et al. 2005 |
| INRACCDDV0033 *(ITGB3)* | AJ874396 | GTGAGTGGTGGTGGTTTCAA | GGCTTGAACTGGCATTCCTA | not polymorph | Chantry-Darmon et al. 2005 |
| INRACCDDV0035 *(CSN3)* | AJ874397 | AACCACCAGGGTCAGCTTC | AGAGTGAGCGAGCGAGAGAC | null | Chantry-Darmon et al. 2005 |
| INRACCDDV0036 *(CD14)* | AJ874398 | CGCACAGACTCAACATCCAG | CCCTACAGGCAGAGGCTTAAT | polymorph | Chantry-Darmon et al. 2005 |
| INRACCDDV0039 *(LCAT)* | AJ874399 | TTGATGGCAGCTTTACATGC | AACCATTGCGACCATCTAGG | polymorph | Chantry-Darmon et al. 2005 |
| INRACCDDV0040 *(ERBB3)* | AJ874400 | CCAGGGTCTGACTACCAGGA | CAGCTGAGGCTCTGTCTGAA | polymorph | Chantry-Darmon et al. 2005 |
| INRACCDDV0042 *(CFTR)* | AJ874401 | AGACCTGCTCCCTTCTTTAGC | ACACACACCCACCCACTCTGGA | no fragment | Chantry-Darmon et al. 2005 |
| INRACCDDV0044 *(ALB)* | AJ874402 | TTTTCATTGAACAAGCTTTGG | CTGCCCTCCCTATTCTTTCC | polymorph | Chantry-Darmon et al. 2005 |
| INRACCDDV0055 *(NOTCH4)* | AJ874403 | TGATTGCTGGGTGCATACAT | GGGGAAAGTTCACCAGGATT | no fragment | Chantry-Darmon et al. 2005 |
| INRACCDDV0057 *(R19)* | AJ874404 | TTCTGGTTCCTGGCTTCATC | TGGAAACAGTTGCCTCTAGGA | not polymorph | Chantry-Darmon et al. 2005 |
| INRACCDDV0058 *(ALOX15)* | AJ874405 | GACAGAGTTCCTGGCTCCTG | TCAGCAGAGCAGAGGGTTTT | not polymorph | Chantry-Darmon et al. 2005 |
| INRACCDDV0061 *(CALR)* | AJ874407 | CCCTGGTGTTGAACCTTTGT | CGACCACAGCTACAGTTCCA | not polymorph | Chantry-Darmon et al. 2005 |
| INRACCDDV0063 *(CYP2C18)* | AJ874409 | GCAGTGTGCCTATGCTTGAA | CCACTCCACCCTATTACCACTG | polymorph | Chantry-Darmon et al. 2005 |
| INRACCDDV0065 *(IGF1)* | AJ874410 | GACCCAGCAGATGGGAGA | TGCAAAAGAACCTTATTGAACATC | not polymorph | Chantry-Darmon et al. 2005 |
| INRACCDDV0068 *(PRNP)* | AJ874412 | CATTTGAGGGGTGAACCAAT | TTGACAGGTAGAGTTATAGACAGTGAA | not polymorph | Chantry-Darmon et al. 2005 |
| INRACCDDV0070 *(TGFA)* | AJ874414 | CCTTGGTTATTGCAGCCATT | AAAGACTTATTTTATTTGAAAGGCAGA | not polymorph | Chantry-Darmon et al. 2005 |
| INRACCDDV0071 *(KRT12)* | AJ874415 | CATCTGGGGAGTGAACCAGT | GATCCCAGATGCTCTGCAT | polymorph | Chantry-Darmon et al. 2005 |
| INRACCDDV0072 *(DSP)* | AJ874416 | AACCAACGGCAAAAGGAAG | AGACGCATTTCCTGCATTTC | not polymorph | Chantry-Darmon et al. 2005 |
| INRACCDDV0074 | AJ874418 | AGGGAGTGAACCAGCAGATG | TTAGGGACATGGCAAGAAGG | polymorph | Chantry-Darmon et al. 2005 |
| INRACCDDV0075 *(SLC6A12)* | AJ874419 | ATTTGCGGAAATGGAAGTT | TTGAACCCTTATTATCCTGACTTCTT | not polymorph | Chantry-Darmon et al. 2005 |
| INRACCDDV0076 *(DLX5)* | AJ874420 | GATCCAGCGGATGGAAGG | CCACTTAACTTGGGAATTCTGC | polymorph | Chantry-Darmon et al. 2005 |
| INRACCDDV0077 *(FSHR)* | AJ874421 | TTCTTTCTCTTATTCATTCATTCATTT | TCAATGACTTCTGAGAACCACAA | polymorph | Chantry-Darmon et al. 2005 |
| INRACCDDV0079 *(AHSG)* | AJ874422 | AGCGTGAGCACTGCTTTATG | GTTTACCTTTTGCCCCACAA | no fragment | Chantry-Darmon et al. 2005 |
| INRACCDDV0080 *(SLC6A12)* | AJ874423 | GTGAACCAGAGGACGGAAGA | CAGGTGGCCTCTTGATCTCT | polymorph | Chantry-Darmon et al. 2005 |
| INRACCDDV0081 *(FXR1)* | AJ874424 | TTGGCAGAAAACATTGCTTG | GAACGGTGCTCCAGTGAGTT | not polymorph | Chantry-Darmon et al. 2005 |
| INRACCDDV0082 *(STAG1)* | AJ874425 | CCAGGACATTGAAGGCAGTT | GAGGTAGGGAAGGGAGAGGA | not polymorph | Chantry-Darmon et al. 2005 |
| INRACCDDV0083 *(DSP)* | AJ874426 | GGGTGAACCAACAGAAGGAA | TCTCTTCCTGGTCTGCCATT | not polymorph | Chantry-Darmon et al. 2005 |
| INRACCDDV0084 *(TGFB3)* | AJ874427 | ATTTGGAGACCGAACCAGTG | TTGAAAATTCCCAAAACACAAA | polymorph | Chantry-Darmon et al. 2005 |
| INRACCDDV0085 *(CYP2C4)* | AJ874428 | AACACTGTGCAAGCACCAAC | TTGCAGCCAATTGAGAAGTG | not polymorph | Chantry-Darmon et al. 2005 |
| INRACCDDV0086 *(GHR)* | AJ874429 | AGCTCCTGGCTTCTTGCTTT | ATGGGATGCCAGTGCTTTAG | not polymorph | Chantry-Darmon et al. 2005 |
| INRACCDDV0087 *(SLC6A12)* | AJ874430 | GATCTGGGACTCCAGAGTGTG | GAACACCGGTCTGGATGG | polymorph | Chantry-Darmon et al. 2005 |
| INRACCDDV0088 *(DMA)* | AJ874431 | CTGGCACCAAGTGTGTGGCATCAT | TTGGTGCAAAACAGGTTGAC | not polymorph | Chantry-Darmon et al. 2005 |
| INRACCDDV0089 | AJ874432 | TTTGAAAGTGTTAGAGGAAGAGAGG | CCAGTCAATGGAAAGTCTTGG | not polymorph | Chantry-Darmon et al. 2005 |
| INRACCDDV0090 | AJ874433 | CGGAAAAACTTACCTCATGTTC | TCCAACTTCTCCATGCCTCTA | not polymorph | Chantry-Darmon et al. 2005 |
| INRACCDDV0091 | AJ874434 | AAATGTGATGCTTGTTCTCAGTT | AACTTTGTTTACAGAAGACCAAACC | polymorph | Chantry-Darmon et al. 2005 |
| INRACCDDV0092 | AJ874435 | GCTTTTGAACCTTCCATGTTG | GAGAAACCAGAAAGCCACTAGAA | polymorph | Chantry-Darmon et al. 2005 |
| INRACCDDV0093 | AJ874436 | GAACCAGCAAAGCAGGAACT | GCCCAAGTTCCGTAGCTAGA | polymorph | Chantry-Darmon et al. 2005 |
| INRACCDDV0094 | AJ874437 | AAGGAGGGCTACACACATTGA | ACCATGCCAGCACTTGATTAC | polymorph | Chantry-Darmon et al. 2005 |
| INRACCDDV0095 | AJ874438 | TGTTTCAGCATGACCACCTC | TAGAAGCACCGAGGAAGGAA | not polymorph | Chantry-Darmon et al. 2005 |
| INRACCDDV0096 | AJ874439 | AACCAATGCCTTTTTCCTCTG | CCAAAACCTCATGGAAGAACA | not polymorph | Chantry-Darmon et al. 2005 |
| INRACCDDV0098 | AJ874440 | GTTGCAGGCATTTGGAGAGTA | AAGGAGAAAGAGACTGAGAGCA | non-specific fragment | Chantry-Darmon et al. 2005 |
| INRACCDDV0099 | AJ874441 | GTGCATCCAGGACAAGTCAG | CTTGAATGATGTTGCGTGGT | non-specific fragment | Chantry-Darmon et al. 2005 |
| INRACCDDV0100 | AJ874442 | GCCTAACCCTGGCTGTTGTA | TGAAAGCAGAATGGCAGAGA | polymorph | Chantry-Darmon et al. 2005 |
| INRACCDDV0101 | AJ874443 | GCCCAAACAGCTGCTATACTG | ACTGCGCAATACACACCTACC | not polymorph | Chantry-Darmon et al. 2005 |
| INRACCDDV0102 | AJ874444 | GCCAAACTTCCTTCAGCCTAT | ACAGCTGTTCGTGCTTTCAGT | polymorph | Chantry-Darmon et al. 2005 |
| INRACCDDV0103 | AJ874445 | CCGTCTTCATTTTCAGCAGTC | ACTGGGAAGCTGGGGATTAT | polymorph | Chantry-Darmon et al. 2005 |
| INRACCDDV0104 | AJ874446 | AGATTTGGCACCCTTGTTCTT | TATTCCCCTGGCAATGAAACT | not polymorph | Chantry-Darmon et al. 2005 |
| INRACCDDV0105 | AJ874447 | AGCCTCCAACTTCTCTTTGC | GGGCAGAGTCCATTCCTTTT | not polymorph | Chantry-Darmon et al. 2005 |
| INRACCDDV0106 | AJ874448 | TCTCTCACTCTCTGCCTCTGC | CATCTTTCGATGGTGTGACCT | polymorph | Chantry-Darmon et al. 2005 |
| INRACCDDV0107 | AJ874449 | CACCTGGTGGTGGAATACTATG | CATTGAACTTTTCCACATCCTGT | not polymorph | Chantry-Darmon et al. 2005 |
| INRACCDDV0108 | AJ874450 | GTTCACTTTTGCTTGCCAGTT | TCTGCAGGCATCCACTAACTT | polymorph | Chantry-Darmon et al. 2005 |
| INRACCDDV0109 | AJ874451 | GCAGTAATAATTAGGCTGAGGAAA | TCAATTCTGTGGCTTCTCTGAA | non-specific fragment | Chantry-Darmon et al. 2005 |
| INRACCDDV0110 | AJ874452 | GTGCTCTGAGCCACAACACT | TGAACACCGACCATATTCCA | polymorph | Chantry-Darmon et al. 2005 |
| INRACCDDV0111 | AJ874453 | CCCAAGAGACCAGGATAGGG | GTTGCAGACATTTGGGGAGT | polymorph | Chantry-Darmon et al. 2005 |
| INRACCDDV0112 | AJ874454 | TTTTGTTTATTTGAAAGGCCAAG | CAGCAGTGAACCACTCTTTCTG | not polymorph | Chantry-Darmon et al. 2005 |
| INRACCDDV0114 | AJ874456 | ACCAATTTTGACTCCCACTCC | GCACTTTGGCACATAGCAGAG | not polymorph | Chantry-Darmon et al. 2005 |
| INRACCDDV0115 | AJ874457 | TACAGGCACAGACACCATGC | TCATGGGATACCCTGATTGAA | polymorph | Chantry-Darmon et al. 2005 |
| INRACCDDV0116 | AJ874458 | GTGGTTTTTCCAGGAGTTTCC | GTGCTCAGAGTAAGGCAGCAG | not polymorph | Chantry-Darmon et al. 2005 |
| INRACCDDV0117 | AJ874459 | CTGAAACGCCCATGTGTCTAT | GTCACAGGGAGGAGACCTGA | not polymorph | Chantry-Darmon et al. 2005 |
| INRACCDDV0118 | AJ874460 | GAAGAGAACTCTCTCCCTGAAAC | AGATTCATAGAGCAAACGAAAAA | not polymorph | Chantry-Darmon et al. 2005 |
| INRACCDDV0119 | AJ874461 | CGGAGAAGAGGTTACCACGA | ATGACCCTGCTTGTCCTCTG | not polymorph | Chantry-Darmon et al. 2005 |
| INRACCDDV0120 | AJ874462 | ACCTATAGGGACCCACACACC | ATGGGCAACTAATGGAAAAGC | polymorph | Chantry-Darmon et al. 2005 |
| INRACCDDV0121 | AJ874463 | TGGACCTAAGACACACATACACAA | GATGGGAGCTCTCTATTGTCG | not polymorph | Chantry-Darmon et al. 2005 |
| INRACCDDV0123 | AJ874464 | GCATTAGCAGGAAGCTGGAG | TAACTTGGTTCAGCCCCACT | polymorph | Chantry-Darmon et al. 2005 |
| INRACCDDV0124 | AJ874465 | TGCAGAACCTTAGAAAGTCATTAAA | TGTTAAAGAATGAAGACTGATTAGA | no fragment | Chantry-Darmon et al. 2005 |
| INRACCDDV0125 | AJ874466 | AAAGGCATTCCAAACACTGG | ACAGGTGTTGCCCAGCTAAA | polymorph | Chantry-Darmon et al. 2005 |
| INRACCDDV0126 | AJ874467 | AGCCATCTGGGGAGTGAAC | AGTGCTTCCCATCTGCTAGTG | polymorph | Chantry-Darmon et al. 2005 |
| INRACCDDV0127 | AJ874468 | GCCTGAGAGTGAAGAGTCAGC | TACCACTACTGCCCCCAAAAC | polymorph | Chantry-Darmon et al. 2005 |
| INRACCDDV0128 | AJ874469 | TCAGTTCCCCAAATACTCACG | TAGGGTGCACTGTGTGTGTGT | not polymorph | Chantry-Darmon et al. 2005 |
| INRACCDDV0129 | AJ874470 | GCCACTGTAATCCAAAAGCTG | TGTGCAATCATTAGGAAACGA | polymorph | Chantry-Darmon et al. 2005 |
| INRACCDDV0130 | AJ874471 | CTGAAATCCTCATGCCAGTGT | GTCCTGCAGAGCACACAGAG | not polymorph | Chantry-Darmon et al. 2005 |
| INRACCDDV0131 | AJ874472 | GGGTTGCTAAGGTTACCGAAT | CCCAAACAGAAAAACCTGACA | non-specific fragment | Chantry-Darmon et al. 2005 |
| INRACCDDV0132 | AJ874473 | GCACGAGTCATTGAGGGATT | TGACCTATTGATAGATGCACACAC | not polymorph | Chantry-Darmon et al. 2005 |
| INRACCDDV0133 | AJ874474 | CAGAAACTACTGGCCCCTCA | CAAGACGGGCCTATCAGAAA | not polymorph | Chantry-Darmon et al. 2005 |
| INRACCDDV0135 | AJ874475 | GGGGCATGGGAATCTTAACT | CCCCAAATATTGCAAAGTGC | not polymorph | Chantry-Darmon et al. 2005 |
| INRACCDDV0136 | AJ874476 | TGCTTATGCTCCTCCTCTTGA | ATCCACCCATCCACACACAC | polymorph | Chantry-Darmon et al. 2005 |
| INRACCDDV0137 | AJ874477 | TACATTGAAAGGAAGCGAAGG | ATGGACGACATTTGTCCCATA | not polymorph | Chantry-Darmon et al. 2005 |
| INRACCDDV0138 | AJ874478 | TTAAGGCACTGCCACAAAAAC | GGCGGTGTCTTTACCCACTAC | not polymorph | Chantry-Darmon et al. 2005 |
| INRACCDDV0139 | AJ874479 | CCAGGAGCACTCTAAGGGACT | CTTCCATGTGTGTGGATTTCA | polymorph | Chantry-Darmon et al. 2005 |
| INRACCDDV0140 | AJ874480 | TCTCTGTTGGCCATCTCCTAA | TCTACTACCCAGCCCCATACC | polymorph | Chantry-Darmon et al. 2005 |
| INRACCDDV0141 | AJ874481 | AATCCTGAGACCAGGGTATGG | TAAACAGCCACGTAAGGCATC | not polymorph | Chantry-Darmon et al. 2005 |
| INRACCDDV0142 | AJ874482 | GGCAGAGTTACACAGAGACAGG | CCTAGCCATTTGGTGAGTGAA | polymorph | Chantry-Darmon et al. 2005 |
| INRACCDDV0143 | AJ874483 | GCATATATCTTTCCCCAGGTTT | AGCTGATGGACTACTTGCAAAA | polymorph | Chantry-Darmon et al. 2005 |
| INRACCDDV0144 | AJ874484 | CCACACCAGAGAGCTTGCTAA | GTGAACCAACAGCAAAGGAAG | non-specific fragment | Chantry-Darmon et al. 2005 |
| INRACCDDV0145 | AJ874485 | GAGAATGAACCAGCAGATGGA | CGTGGCCTCCACTATACTCAG | polymorph | Chantry-Darmon et al. 2005 |
| INRACCDDV0146 | AJ874486 | GACTGAACCAGCAGATGGAAG | CCTATTAAAATCAGCCCCACA | polymorph | Chantry-Darmon et al. 2005 |
| INRACCDDV0147 | AJ874487 | CTAATCCAACCATGCCACAA | CAGGTGACAGAATGTGTGTGTG | non-specific fragment | Chantry-Darmon et al. 2005 |
| INRACCDDV0148 | AJ874488 | CCTGAGCTTAAAATGCGAAA | CTTTCCTGGTTGATGCCAGTA | polymorph | Chantry-Darmon et al. 2005 |
| INRACCDDV0149 | AJ874489 | CCAGAGCTCCTAGGACCAAGA | TTGGAAAACGAGTGAGAGGAG | not polymorph | Chantry-Darmon et al. 2005 |
| INRACCDDV0150 | AJ874490 | CTTGCTGCAAATGACAGGATT | CGTGTTTATAGCAGCCCTGTTC | not polymorph | Chantry-Darmon et al. 2005 |
| INRACCDDV0151 | AJ874491 | AACTCAGCCAAATGGAGAATG | CTAGGGGGTAGCTCTGGATTG | polymorph | Chantry-Darmon et al. 2005 |
| INRACCDDV0152 | AJ874492 | CCAGGCTCTTGCCTCTTATCT | ACTCTCCTCTCGCCTCTCACT | not polymorph | Chantry-Darmon et al. 2005 |
| INRACCDDV0153 | AJ874493 | AATGCCCCTCAGTTCTTCTTC | CAATTAGGGAGTGAACCAGCA | polymorph | Chantry-Darmon et al. 2005 |
| INRACCDDV0154 | AJ874494 | CAAACTGTAATTGTGTGTG | TGGATGAACCATTAAGAGCAG | not polymorph | Chantry-Darmon et al. 2005 |
| INRACCDDV0155 | AJ874495 | CCTGAGTGTTTGGACTGGAAG | TCCTCATCAAACAGACCCAAT | polymorph | Chantry-Darmon et al. 2005 |
| INRACCDDV0156 | AJ874496 | ATAAGGTTGCCAAGGAAATGC | GTGTTAAGCCCATGCTCATTC | not polymorph | Chantry-Darmon et al. 2005 |
| INRACCDDV0157 | AJ874497 | AACACTTGCCCCTCTTTTCAT | CAGGTTGTGGGAGTTCTTGTC | null | Chantry-Darmon et al. 2005 |
| INRACCDDV0158 | AJ874498 | TCCCCTACTCTGTCATTCTGC | AGCTCTGGAAAGGAGAAGAGG | polymorph | Chantry-Darmon et al. 2005 |
| INRACCDDV0159 | AJ874499 | GATCACCTACTGAAGACACACAAC | CCCTACTTGGCAGATTCTTAAC | polymorph | Chantry-Darmon et al. 2005 |
| INRACCDDV0160 | AJ874500 | CCAGAACTTTCCAGACAGCAT | CCTAAAACGCTGTCATCCTGA | not polymorph | Chantry-Darmon et al. 2005 |
| INRACCDDV0162 | AJ874501 | CAATCAAACAAAGTCTGCAAGG | ATGCTCATTCAACCCTCCATA | polymorph | Chantry-Darmon et al. 2005 |
| INRACCDDV0163 | AJ874502 | AGGTGCTCCCAGACAGCTTTA | CCTCCCCAAGTCTTTCCTAGA | polymorph | Chantry-Darmon et al. 2005 |
| INRACCDDV0164 | AJ874503 | CTGCGGAAGTCAGGAGATTTA | TGAACCAGCAAATGGAAGAAC | polymorph | Chantry-Darmon et al. 2005 |
| INRACCDDV0165 | AJ874504 | TTGACCTCCAACTCTTCCAGA | TTATGTTCGTGCAGATTGCTG | polymorph | Chantry-Darmon et al. 2005 |
| INRACCDDV0166 | AJ874505 | CTGGTCTGCACCTTAGTCTCC | AGATGTTGAGGAGCATGGTTG | not polymorph | Chantry-Darmon et al. 2005 |
| INRACCDDV0167 | AJ874506 | TAGTTGCTTGCCTGCTGAACT | GGCAGGTACTCAAGCAATCCT | not polymorph | Chantry-Darmon et al. 2005 |
| INRACCDDV0168 | AJ874507 | ACACAAAACACGAAGGCACAT | GCAAGCAGCTGAAAGTACCAC | polymorph | Chantry-Darmon et al. 2005 |
| INRACCDDV0169 | AJ874508 | AGCACCCACATGATGAAAGTC | GAGCGACAAATCCAGCTCAT | polymorph | Chantry-Darmon et al. 2005 |
| INRACCDDV0171 | AJ874509 | TCCGATAGCCACGTTAAAAGA | GCGGCACCTAAACTCAGAAC | not polymorph | Chantry-Darmon et al. 2005 |
| INRACCDDV0172 | AJ874510 | ATTGTCCAAAACATGCTCGTG | TTCCACTGAGAAAGCGTGAGT | polymorph | Chantry-Darmon et al. 2005 |
| INRACCDDV0173 | AJ874511 | GCAGAACATGGTCAGACATCA | GGCATGAGGACGAGATAGAAA | polymorph | Chantry-Darmon et al. 2005 |
| INRACCDDV0174 | AJ874512 | CTCACGATCACATCGAGACTG | GAGCCTCACTGTCCCTCATCT | no fragment | Chantry-Darmon et al. 2005 |
| INRACCDDV0175 | AJ874513 | ACAAGGGGTAATCAGGGTTTG | CCTGCCTCACATGAAATGAAT | non-specific fragment | Chantry-Darmon et al. 2005 |
| INRACCDDV0176 | AJ874514 | CTTGGAGAAAGGCCTGAGAAA | TAGCTCTCTGCCAACACCACT | polymorph | Chantry-Darmon et al. 2005 |
| INRACCDDV0177 | AJ874515 | TGTGTTTGTGTGGGAAAGTGA | TTCTAAATGCTTCAGCCTTGC | polymorph | Chantry-Darmon et al. 2005 |
| INRACCDDV0178 | AJ874516 | TCTATCCAAGGCAGACAGACG | ACCCAGATGAGAGACCTGGAT | not polymorph | Chantry-Darmon et al. 2005 |
| INRACCDDV0179 | AJ874517 | AATTCACCTCTGCACTTCTGC | TCTCTGCCTCTCCTTCTCTCC | not polymorph | Chantry-Darmon et al. 2005 |
| INRACCDDV0180 | AJ874518 | TGCTTAACACCTCAGAGAAGC | CTCTGCATGTCTCCTCATAAAATG | non-specific fragment | Chantry-Darmon et al. 2005 |
| INRACCDDV0181 | AJ874519 | TGTCTTTCCCTCTCACTGTCTG | TTGGACTGTAAACCAGTGGAAA | non-specific fragment | Chantry-Darmon et al. 2005 |
| INRACCDDV0182 | AJ874520 | AACCTAGTTGGGAGCAGTGTG | GATTCATAGCTGTGCCTTTCAA | polymorph | Chantry-Darmon et al. 2005 |
| INRACCDDV0183 | AJ874521 | CCTCTGCTAACCACCGTTTTA | CATATGAGGGGTGAACCAATG | polymorph | Chantry-Darmon et al. 2005 |
| INRACCDDV0184 | AJ874522 | CAGAAGGGGTAAAGTCATGTTG | CACCAAGCTTTTGTGTGTTGA | polymorph | Chantry-Darmon et al. 2005 |
| INRACCDDV0185 | AJ874523 | GTTCACTCAAAGTAACTGACAAGCA | TCCATTCAGCAACCAATGAA | polymorph | Chantry-Darmon et al. 2005 |
| INRACCDDV0186 | AJ874524 | GAAACAGCCCAGGAAAATATGA | TAGCCTGCAGAAATGGAACAT | not polymorph | Chantry-Darmon et al. 2005 |
| INRACCDDV0187 | AJ874525 | CAGCTCTCCAGAGCACAAAAC | AGAAGAGGGGGTGACCTACAG | not polymorph | Chantry-Darmon et al. 2005 |
| INRACCDDV0188 | AJ874526 | GGCCACCCAGTTGTTTATTCT | AATAGTGGTGGCCCAGATAATG | polymorph | Chantry-Darmon et al. 2005 |
| INRACCDDV0189 | AJ874527 | TTGGGTTGGCTGTATTAGTGC | GTGGGAGAGCAGATGCATAGA | not polymorph | Chantry-Darmon et al. 2005 |
| INRACCDDV0190 | AJ874528 | GCCAATACCAAAAACAGCAAC | TGGGAGAGCTAAGAAAAGTCG | non-specific fragment | Chantry-Darmon et al. 2005 |
| INRACCDDV0191 | AJ874529 | GGTGTTTCCTCGCAGTGTGTA | GCCGCATGAGCTTTTGTAATA | polymorph | Chantry-Darmon et al. 2005 |
| INRACCDDV0192 | AJ874530 | TGCAATAGGTGGAGGCTTAGA | TCCACAGAGGAGATATAGTGGTCTT | polymorph | Chantry-Darmon et al. 2005 |
| INRACCDDV0193 | AJ874531 | CCATTTGGGGAGTAAACCAGT | CTCTTCTGTGGCGAGATGTGT | polymorph | Chantry-Darmon et al. 2005 |
| INRACCDDV0194 | AJ874532 | CAGTTGGGAAGGAAACCATC | GATTTATGTGAGAGGCAGAGTTACA | polymorph | Chantry-Darmon et al. 2005 |
| INRACCDDV0195 | AJ874533 | CATGTCTGTAAACACACACACACA | TGACGTCATGCACAGCTTAAA | not polymorph | Chantry-Darmon et al. 2005 |
| INRACCDDV0196 | AJ874534 | CCAAGCAGCAAGGAAACATTA | CTCGACTAGACCTCTGGGTCA | non-specific fragment | Chantry-Darmon et al. 2005 |
| INRACCDDV0197 | AJ874535 | GAACCAGAGGAACAGGGAGAG | AGCCTTCGAAGTTGAGGAGTG | not polymorph | Chantry-Darmon et al. 2005 |
| INRACCDDV0198 | AJ874536 | GTGCTGACAGGCATGGAATAG | CCTTGCCTGATTCAGATTTGT | polymorph | Chantry-Darmon et al. 2005 |
| INRACCDDV0200 | AJ874537 | CCTGGTGCTGCAAAAGTTATT | TAGGCCATTTGGGGATGAAT | not polymorph | Chantry-Darmon et al. 2005 |
| INRACCDDV0201 | AJ874538 | AGGCAGGTAAGGGGGAAAG | GCATTTGGGGAAGTAACCAGT | polymorph | Chantry-Darmon et al. 2005 |
| INRACCDDV0202 | AJ874539 | TTTCTAAGCTCCTGGCTTTGG | GGAAAGAATTACACACACACACAC | non-specific fragment | Chantry-Darmon et al. 2005 |
| INRACCDDV0203 | AJ874540 | GATCCAGTCATGGTGTGTGTG | TAGGGCTGGGTTTTTATCTGG | polymorph | Chantry-Darmon et al. 2005 |
| INRACCDDV0204 | AJ874541 | TGATGCACGTAGAACAAGCAC | TCGAGAATCAAAACCGTTGAC | null | Chantry-Darmon et al. 2005 |
| INRACCDDV0205 | AJ874542 | CAGGGATTCCATCAATGACAC | GGCCCCTGGATTAGTTTTCTA | not polymorph | Chantry-Darmon et al. 2005 |
| INRACCDDV0206 | AJ874543 | GAGTGAACCAGTGGATGGAAA | CTGGAGGGGTTTTCTTAATCTT | not polymorph | Chantry-Darmon et al. 2005 |
| INRACCDDV0208 | AJ874544 | GTGAACCAGCAGATGGAAGG | CATCACAGGTGGTGCTTTTT | no fragment | Chantry-Darmon et al. 2005 |
| INRACCDDV0211 *(HSA3)* | AJ874545 | CACAGCCCTACACACACACC | CTGGTCTGCAGTGAAAGCAA | polymorph | Chantry-Darmon et al. 2005 |
| INRACCDDV0212 | AJ874546 | GGGGAGGGAAAGAAAGAGG | TGGCTTCCTATTGGCTCATC | not polymorph | Chantry-Darmon et al. 2005 |
| INRACCDDV0213 *(PRKCB1)* | AJ874547 | CCCACATAGGAGACCTGGAA | AGGGAGAGAAAGAGACCAGACA | polymorph | Chantry-Darmon et al. 2005 |
| INRACCDDV0214 *(EIF3S8)* | AJ874548 | CAGATGGTGGCCTTACCA | GGAGTGAACCACGGAAGACT | polymorph | Chantry-Darmon et al. 2005 |
| INRACCDDV0215 | AJ874549 | CACCGTTTCCCATAGAGTCC | GCTCCTGGCTTAGGATTAGC | not polymorph | Chantry-Darmon et al. 2005 |
| INRACCDDV0216 | AJ874550 | CCAAGTGCCCAGTCCTAGAG | CACTCCCAAGTTCAGGCTTC | no fragment | Chantry-Darmon et al. 2005 |
| INRACCDDV0217 *(GMFB)* | AJ874551 | GGGGAGTGGAAGACCTCTCT | CTCCCTCCACCTGAACATTT | polymorph | Chantry-Darmon et al. 2005 |
| INRACCDDV0218 *(ARFGEF1)* | AJ874552 | CATTTGGGGGAGTGAATCAG | GCAGAGTTACAGGGACAGAGG | polymorph | Chantry-Darmon et al. 2005 |
| INRACCDDV0219 *(MYH11)* | AJ874553 | GGGGAGTGAACCAGCAGAT | ATGCAGCCATCCTGACACTT | polymorph | Chantry-Darmon et al. 2005 |
| INRACCDDV0221 *(GPR37)* | AJ874555 | GGGTCCAAACAGAACAGGAG | GAAACGGATGTCCCAGCTTA | polymorph | Chantry-Darmon et al. 2005 |
| INRACCDDV0224 | AJ874557 | GGCCAATTGAGAAGTGAACC | GGTCTCCCACAAGTCACTGAG | no fragment | Chantry-Darmon et al. 2005 |
| INRACCDDV0225 *(ASPH)* | AJ874558 | AAACTGGAGAGGGGAAGCAG | AAGGCTTCTCTCAGGGATGA | polymorph | Chantry-Darmon et al. 2005 |
| INRACCDDV0228 | AJ874561 | ACTCCCAGCCTCAGCTGTT | ATGCTGCTGTGGGACAGACT | polymorph | Chantry-Darmon et al. 2005 |
| INRACCDDV0229 | AJ874562 | CTGTCCACTCTGCCTGTCAA | GCCAGGAAAATGAAATTGG | not polymorph | Chantry-Darmon et al. 2005 |
| INRACCDDV0230 | AJ874563 | CAATTGGGGAGTGAACCAGT | GGGAAAATCTTGACATTAGCTC | polymorph | Chantry-Darmon et al. 2005 |
| INRACCDDV0231 *(GPR37)* | AJ874564 | CCTACAGCTGGGAACACACA | TCAAGAGCAAGGGGAAGAGA | polymorph | Chantry-Darmon et al. 2005 |
| INRACCDDV0232 | AJ874565 | ATCATGGGTATTGGGGAGTG | AGCCAGAAAGACAGACACAGG | not polymorph | Chantry-Darmon et al. 2005 |
| INRACCDDV0233 | AJ874566 | ACCAACGGCAAAGGAAGAC | AGCAGGTGTCACAAGCAGTG | non-specific fragment | Chantry-Darmon et al. 2005 |
| INRACCDDV0234 *(NDEL1)* | AJ874567 | TGGTCCTGAATGGAAACTCC | AGTGAACCAGTGGATGGAAGA | polymorph | Chantry-Darmon et al. 2005 |
| INRACCDDV0235 | AJ874568 | GGAAACTGGTGGGAAAGTTG | TAAGTCCAGGATGCAGCAGA | not polymorph | Chantry-Darmon et al. 2005 |
| INRACCDDV0236 *(TJP2)* | AJ874569 | GCGGATGGAAGACCTCAAG | TCTCCCCTCCACCTTACCTT | polymorph | Chantry-Darmon et al. 2005 |
| INRACCDDV0237 *(NNT)* | AJ874570 | GAACCAGCAGATGGAAGCTC | GGAGGAAGGAGTGGATGTCA | polymorph | Chantry-Darmon et al. 2005 |
| INRACCDDV0238 | AJ874571 | CCCGTGTCTGTACCAAGTCA | GTGAGCCGAACCTCAATAGC | not polymorph | Chantry-Darmon et al. 2005 |
| INRACCDDV0239 | AJ874572 | GGTGAACCAGCAAGTGGAA | CCTCCTCCCCCTCTTCTTCT | non-specific fragment | Chantry-Darmon et al. 2005 |
| INRACCDDV0240 *(DAPK1)* | AJ874573 | ATGCTTGAGACCCCGAATC | TCCCCTGACTGTATCCCATC | polymorph | Chantry-Darmon et al. 2005 |
| INRACCDDV0241 *(TIAM1)* | AJ874574 | ATCAAGGACAAGGCAGCAAT | TGCCTTCTAGGAAGCCAGAAT | polymorph | Chantry-Darmon et al. 2005 |
| INRACCDDV0243 | AJ874576 | AAGTACTTGAGCCCCTGCAC | AGAGGGAGGGAGTGAGGAAG | non-specific fragment | Chantry-Darmon et al. 2005 |
| INRACCDDV0247 | AJ874578 | TGGATCATGTCATTGGCTGT | CAGTCCTAACAGGGCTGGAA | no fragment | Chantry-Darmon et al. 2005 |
| INRACCDDV0248 *(PMCH)* | AJ874579 | CTCTGTAATCTGCCTCCCAAA | GACATCTGGAAAGTGAACAAGC | polymorph | Chantry-Darmon et al. 2005 |
| INRACCDDV0249 | AJ874580 | AAGAGACCTCTGCCTTGGTG | CTGTTTGGAACTATCTGGAGCA | not polymorph | Chantry-Darmon et al. 2005 |
| INRACCDDV0251 | AJ874582 | AGCCATCTGGGGAGTGAAC | ATCTCATACGGGTGCCAGTT | non-specific fragment | Chantry-Darmon et al. 2005 |
| INRACCDDV0252 *(HTR3B)* | AJ874583 | GGGCATTTAGGAGTGAACCA | GAAACCTGGATGGGATTCCT | polymorph | Chantry-Darmon et al. 2005 |
| INRACCDDV0253 | AJ874584 | CCTCCCTCTCTTCCTCCTTC | AGCAAGTTGTTACCCGCTGT | not polymorph | Chantry-Darmon et al. 2005 |
| INRACCDDV0256 *(MSN)* | AJ874586 | GTGAATCAGCGGAATGGAAG | CATGGGAACAGTCTGTGTGG | polymorph | Chantry-Darmon et al. 2005 |
| INRACCDDV0257 *(PARVA)* | AJ874587 | GAACCAGCGGATGGAAGA | GTCCTTTCGTTTTTCACTTCAC | no fragment | Chantry-Darmon et al. 2005 |
| INRACCDDV0258 *(MINPP1)* | AJ874588 | AGTGGACCATCAGACGGAAG | GGGTGGTTGTGTTAGGTCCA | polymorph | Chantry-Darmon et al. 2005 |
| INRACCDDV0259 *(TJP2)* | AJ874589 | ATCCTCTCTCCTTTGCATGG | TCCAGTGGTCTGCTTTTTCA | not polymorph | Chantry-Darmon et al. 2005 |
| INRACCDDV0260 *(RNGTT)* | AJ874590 | AGCCACACAGGGAGAGGAG | TTGTGGCACTGACCATTTTG | not polymorph | Chantry-Darmon et al. 2005 |
| INRACCDDV0263 *(DAPK1)* | AJ874593 | AGCTTGAGTCCCTTGAGATCC | GGAAGACACACACTGAGAGAGA | no fragment | Chantry-Darmon et al. 2005 |
| INRACCDDV0269 *(TJP2)* | AJ874595 | GGGTGAACCAACAGAAGGAA | TCTGGCAGATTGCACACTTT | polymorph | Chantry-Darmon et al. 2005 |
| INRACCDDV0270 *(BCAS2)* | AJ874596 | CCTTTGGTTTGGGTCTTCCT | TGTGGCCATTTGAGAAGTGA | polymorph | Chantry-Darmon et al. 2005 |
| INRACCDDV0271 *(HTR3B)* | AJ874597 | CAGTGAAGCCTTGGAGGAAC | TAGCAAAGCGAAGGTGAAGG | polymorph | Chantry-Darmon et al. 2005 |
| INRACCDDV0272 *(PTCH)* | AJ874598 | CACAGAGTGCGGACACCTTA | CCATGGGTGGGTCAATATCT | not polymorph | Chantry-Darmon et al. 2005 |
| INRACCDDV0273 *(GALNT1)* | AJ874599 | CCATGCAGCTATTTGGGAGT | GGGACCACTTCCCAGTTGTA | not polymorph | Chantry-Darmon et al. 2005 |
| INRACCDDV0274 *(ITIH3)* | AJ874600 | GTGTGTGAGAGGTGGTGGTG | ATCAGGGTCCAGCCATGC | polymorph | Chantry-Darmon et al. 2005 |
| INRACCDDV0275 *(MSN)* | AJ874601 | ACCCATGTGGGAGACCTGTA | TCCCTCTCTCACTGTCACCA | not polymorph | Chantry-Darmon et al. 2005 |
| INRACCDDV0276 | AJ874602 | GAGACCAGGAAAAGCACCTG | TTGACAGGCAGAGTGGACAG | non-specific fragment | Chantry-Darmon et al. 2005 |
| INRACCDDV0278 *(AQP7)* | AJ874603 | AGTGGCCAATAGAGGGTGAA | GGAGGTCCACAGGAGTGTGT | not polymorph | Chantry-Darmon et al. 2005 |
| INRACCDDV0279 | AJ874604 | TCATGGGAGACCTGGATGAT | GGGGAGTGAACCAGCAGATA | polymorph | Chantry-Darmon et al. 2005 |
| INRACCDDV0280 | AJ874605 | ACCTGTCAGAGCCAAGGATG | GGACATACATGCTGCCACAC | polymorph | Chantry-Darmon et al. 2005 |
| INRACCDDV0282 | AJ874606 | CCAGCCCCTGAACTCTTTTT | TTCCAGATTGCAGGTTGTGA | polymorph | Chantry-Darmon et al. 2005 |
| INRACCDDV0283 | AJ874607 | ATGAGTAGAGTGTATGTATA | AAAGGGCAATTGGGAAGAGT | no fragment | Chantry-Darmon et al. 2005 |
| INRACCDDV0285 | AJ874608 | GGTGTTCTCTCTGTCCCAGAA | TGGGAAGTGAACCAGCAAAT | not polymorph | Chantry-Darmon et al. 2005 |
| INRACCDDV0286 | AJ874609 | GTTTTGCTCTGGAGGTGGAG | CAGGGAGAGTGGATGTGGTT | polymorph | Chantry-Darmon et al. 2005 |
| INRACCDDV0287 | AJ874610 | GAGAATGTGCGCTGTTTCAA | CAGTGGAGACACACCCTCAA | polymorph | Chantry-Darmon et al. 2005 |
| INRACCDDV0288 | AJ874611 | AATCTTTTGGCCTCCTGTCC | TTTTGTTGCTCCAGGCTCTT | polymorph | Chantry-Darmon et al. 2005 |
| INRACCDDV0289 | AJ874612 | AGAATGGGTGAGTGGTTAGGC | ACTAGCGGATGGAAGACCTGT | polymorph | Chantry-Darmon et al. 2005 |
| INRACCDDV0290 | AJ874613 | GCGGTCTGGAAAAAGTGTGT | TGTGCTGTTGTGGCTTAACG | polymorph | Chantry-Darmon et al. 2005 |
| INRACCDDV0291 | AJ874614 | CAAGAAGCCCTGTGGAATGT | GAGCAGTTCTGCCTCTGGTC | polymorph | Chantry-Darmon et al. 2005 |
| INRACCDDV0292 | AJ874615 | ACCTGGCTTACCTTGCATTG | GACCTGCCTCCCTCATCTCT | not polymorph | Chantry-Darmon et al. 2005 |
| INRACCDDV0293 | AJ874616 | GGAACAGCCTTTTCTCCTCA | CATTTGGGGAAGGAACCAGT | polymorph | Chantry-Darmon et al. 2005 |
| INRACCDDV0294 | AJ874617 | AAAGCAGAGTGGCAGAGAGG | AAAAGATGGCCCAAGAACCT | polymorph | Chantry-Darmon et al. 2005 |
| INRACCDDV0296 *(NPC1)* | AJ874618 | GAAAGGGAAAGTCTGGAATGC | TGAAGCTCTGCCTCTGTTAGG | polymorph | Chantry-Darmon et al. 2005 |
| INRACCDDV0297 | AJ874619 | CACTGCAGGCAGAAGATTAGC | GGCAACTTAACTGTTGCGTGT | polymorph | Chantry-Darmon et al. 2005 |
| INRACCDDV0298 | AJ874620 | GCCGTCTACCCAAGTCTCAA | TGAACCAGTGGATGGAAACA | polymorph | Chantry-Darmon et al. 2005 |
| INRACCDDV0299 | AJ874621 | TCTCTCTGGCCCACCATAAC | AGGGAGTGAACCAGTGGATG | polymorph | Chantry-Darmon et al. 2005 |
| INRACCDDV0300 | AJ874622 | TGCTGGTGCCTCCAATTAAC | GGAAGCCGAAAATGAGTAGTG | not polymorph | Chantry-Darmon et al. 2005 |
| INRACCDDV0301 | AJ874623 | GCTGAAAGAACAAACCTTGG | GTTGCAGCCAATTACGGAGT | not polymorph | Chantry-Darmon et al. 2005 |
| INRACCDDV0302 | AJ874624 | GATGGGTTTGCTCACACCTT | AGCTCAGAGGCCCGTAGTG | polymorph | Chantry-Darmon et al. 2005 |
| INRACCDDV0303 *(SLC4A4)* | AJ874625 | TTCTAACTGCCTTCAGGATGC | TTGAGCACATTAGGCTCTTCC | polymorph | Chantry-Darmon et al. 2005 |
| INRACCDDV0304 *(EGFR)* | AJ874626 | GGGGGTCACTTCCCTTTTT | CATGCTTTTGCCAATGTCAC | polymorph | Chantry-Darmon et al. 2005 |
| INRACCDDV0305 *(EGFR)* | AJ874627 | GAGGGTGTCCTAAGCAGTCTT | AACCAGCAGATGGAAGATCG | not polymorph | Chantry-Darmon et al. 2005 |
| INRACCDDV0306 *(GC)* | AJ874628 | GGCATTCGGATCAAAGTTAC | GCCTTGTCCATGGTAGCTG | polymorph | Chantry-Darmon et al. 2005 |
| INRACCDDV0307 *(SLC4A4)* | AJ874629 | TTCCCACTCCACTCTCCTTC | GCACTGCTTTTGGGAAGATT | not polymorph | Chantry-Darmon et al. 2005 |
| INRACCDDV0308 | AJ874630 | GGAAGGCCATGCATTCTG | GGTGAACCAACGGTAAAGGA | not polymorph | Chantry-Darmon et al. 2005 |
| INRACCDDV0309 *(MYH3)* | AJ874631 | CAGAGAGAAGGAGAGGCAGAA | GCCGTTGCAGCCAGTTAC | no fragment | Chantry-Darmon et al. 2005 |
| INRACCDDV0310 *(PTAFR)* | AJ874632 | TCTCACTGATCACTCTGCCTGT | TGTGGAAGTGCCCCAAAT | polymorph | Chantry-Darmon et al. 2005 |
| INRACCDDV0311 *(CALU)* | AJ874633 | GAACCAGCAGATGGAAGACC | TCCCTTCGTCCTCTGTCAAT | polymorph | Chantry-Darmon et al. 2005 |
| INRACCDDV0313 *(HES1)* | AJ874634 | TGTGATACCCCGGAGTGTTT | TCGCTTGACTTCTTGGGTCT | polymorph | Chantry-Darmon et al. 2005 |
| INRACCDDV0314 *(PRNP)* | AJ874635 | CCTGTAGTAATACCGCCCACA | CGGGAGACAGGTATGGAGTT | polymorph | Chantry-Darmon et al. 2005 |
| INRACCDDV0317 *(ORL1)* | AJ874637 | GGCCTTATCCCTATGCCAGT | GTCCAGCTCCCATCATTGTG | not polymorph | Chantry-Darmon et al. 2005 |
| INRACCDDV0318 *(CGA)* | AJ874638 | TGCACCATATGTGGTTGTCC | AGATGGAGTTCCTGGCTCCT | non-specific fragment | Chantry-Darmon et al. 2005 |
| INRACCDDV0320 *(SLN)* | AJ874640 | TAGGGAGTGAGCCAATGGAC | GGGTAGAGTGACACAGAGAGGAA | polymorph | Chantry-Darmon et al. 2005 |
| INRACCDDV0322 *(SLN)* | AJ874641 | GGCCCTCTAATAAGGGATGC | CTTCAATTCAGCCCAGCTCT | not polymorph | Chantry-Darmon et al. 2005 |
| INRACCDDV0323 *(PROC)* | AJ874642 | CATTTGGGGAGTGAACCAGT | AGCTCTGGGGTCAGAGGTTT | polymorph | Chantry-Darmon et al. 2005 |
| INRACCDDV0325 *(PRNP)* | AJ874643 | AGTTCCAGCTGCTCCTCTTCT | GCCTGGGAGAGCAGTAGAAA | not polymorph | Chantry-Darmon et al. 2005 |
| INRACCDDV0326 *(SRY)* | AJ874644 | AGCAGGTGGTGGCTCAAGTA | GTCCTTGGACCCATGTCAGA | no fragment | Chantry-Darmon et al. 2005 |
| INRACCDDV0327 *(RPS3)* | AJ874645 | GCCCGTCAAGAGAAACACAT | CTGGGGAGTAAACCAGTGGA | not polymorph | Chantry-Darmon et al. 2005 |
| INRACCDDV0328 *(PSAT1)* | AJ874646 | TGTGCAACTTTTGGCTCGT | CACAGGTGACACAGAAACCAA | not polymorph | Chantry-Darmon et al. 2005 |
| INRACCDDV0329 | AJ874647 | CCACGTTCTGACAAAATGGA | GGAAACAACCCCATTATCAACAG | non-specific fragment | Chantry-Darmon et al. 2005 |
| INRACCDDV0331 *(C2ORF25)* | AJ874649 | CCTACTCCCCAGTCAGGAACT | CATTTGGGGAGCAAACCA | no fragment | Chantry-Darmon et al. 2005 |
| INRACCDDV0333 *(PRNP)* | AJ874650 | GAGCAGGAGGAAGAAACCAG | GGGAGGCACACAGACACATA | polymorph | Chantry-Darmon et al. 2005 |
| INRACCDDV0334 *(RPS4X)* | AJ874651 | GCAAATCTGGTGCACAAAGAAG | TCGCTGACCAAAGGTTAGAA | not polymorph | Chantry-Darmon et al. 2005 |
| INRACCDDV0335 *(SIAH2)* | AJ874652 | CAGGGGGAGAGAGAAACAGA | CTCTGCTATGGCCTGGAAAG | not polymorph | Chantry-Darmon et al. 2005 |
| INRACCDDV0336 *(PROC)* | AJ874653 | GTTGTGGGCATTTGAGGAGT | GGCGTTAACCTGCTTTACCA | polymorph | Chantry-Darmon et al. 2005 |
| INRACCDDV0337 *(SIAH2)* | AJ874654 | GTTGCAGCCATTTCTGGAGT | ACAGCTGTGGGAAGGACATC | polymorph | Chantry-Darmon et al. 2005 |
| INRACCDDV0338 *(TNFRSF6)* | AJ874655 | TGGAAAGCATGGCTATGACA | TGTTGGCACTTGGGTTACAA | not polymorph | Chantry-Darmon et al. 2005 |
| INRACCDDV0339 *(ENO3)* | AJ874656 | CCACCTAGTCACTCCCTCCA | TGCACCAGTTACAGGCTGAG | not polymorph | Chantry-Darmon et al. 2005 |
| INRACCDDV0340 *(NCOA6)* | AJ874657 | CATTCACCAGAGGCCTGATT | GAGTAAGCCAATGGGTGGAA | polymorph | Chantry-Darmon et al. 2005 |
| INRACCDDV0341 *(TPT1)* | AJ874658 | GGCTCCTTACACACACCTAAGC | CTCCTTGATGGTGGCAAA | polymorph | Chantry-Darmon et al. 2005 |
| INRACCDDV0342 | AJ874659 | CACACCATCGATTTGACCAG | TGGGGAGTGAACCACTAGAT | not polymorph | Chantry-Darmon et al. 2005 |
| INRACCDDV0344 *(MAPRE2)* | AJ874660 | GGAATCTGCACCACCAAGAT | AGGTGGGTGGCTATGTTCAG | polymorph | Chantry-Darmon et al. 2005 |
| INRACCDDV0345 *(PSAT1)* | AJ874661 | GGGAAGTCAATCGCAGATCA | TCATGGAATCTCCGGACTGT | polymorph | Chantry-Darmon et al. 2005 |
| INRACCDDV0346 *(CRP)* | AJ874662 | ATGGAGGGTTTCTCCCCAAT | CAGCCAAATAGCCACAGTCA | not polymorph | Chantry-Darmon et al. 2005 |
| INRACCDDV0347 *(EPO)* | AJ874663 | CCACAACACCAGCACCAATA | AACTGGGGAGTGAACCAGTG | non-specific fragment | Chantry-Darmon et al. 2005 |
| INRACCDDV0349 *(AOAH)* | AJ874664 | GACCCCTTTACCCCAACCTA | ACTGCTGTCTCCCATGCTCT | not polymorph | Chantry-Darmon et al. 2005 |
| INRACCDDV0350 *(RPS3)* | AJ874665 | TGTTTGCTCTCGCTCTCTCA | GTCACAACCACTGTGCCAAG | not polymorph | Chantry-Darmon et al. 2005 |
| INRACCDDV0351 *(MMP1)* | AJ874666 | CAGCCAGGCATCTCTCTCTC | ACAAGGTGGTGGTACCGAAG | no fragment | Chantry-Darmon et al. 2005 |
| INRACCDDV0357 *(BRIX)* | AJ874670 | GCCAATTGGGGAGTGAACTA | CCCATCTGGGTCTGATGTTT | not polymorph | Chantry-Darmon et al. 2005 |
| INRACCDDV0358 | AJ874671 | GGGGCATCTCGATACTCTCA | CCTTCTACCGCTCCCACATA | not polymorph | Chantry-Darmon et al. 2005 |
| INRACCDDV0359 *(CXCR4)* | AJ874672 | ATTATGGCCATTTGGGGAGT | AGAGGAGCAACTGGGACAGA | non-specific fragment | Chantry-Darmon et al. 2005 |
| OCAS1CG *(CSN1S1)* | AY284844 | AGAGAGAGGGAGATGCACACA | TTTGGATAGGCCCAGATCTG | polymorph | vanHaeringen et al. 1996 |
| OCBGLX | V00883 | TCTAGGAAGAAGCTTTATCCCTC | GTTTTCTCATCAGAAATCCCACC | not polymorph | vanHaeringen et al. 1996 |
| OCCRP *(CRP)* | M14538.1 | AGGGTACGCCAGAGATTTGAAC | TACCCACGAGTCATGGACAGAT | polymorph | vanHaeringen et al. 1996 |
| OCELAMB *(ELAMB)* | M91004 | AGTCACATTTGGCATTTCGTGA | TCCTTTGAATTTAGGATCCACAGC | polymorph | vanHaeringen et al. 1996 |
| OCLR1 | D10111 | GCTACTGGCTTCAGATTGGC | AGGCAGACCTCAAAAAAATAAGG | no fragment | vanHaeringen et al. 1996 |
| OCMCP1AB (*MCP1AB*) | M28073.1 | GTGGAACTCAAGCATGCAGA | GACATTTTATCGGGGCACAC | no fragment | vanHaeringen et al. 1996 |
| OCPRG5 *(PRG5)* | M14547.1 | ATTGTGAGTAGCAGGGGTGG | CATAAGATTCCCCAAAGACTGC | polymorph | vanHaeringen et al. 1996 |
| OCRLADF4 *(RLADF4)* | X60986 | TTCCTTTCTGTCCTGAGACCATG | GCAGTTGTGTGGAAATTTGGC | polymorph | vanHaeringen et al. 1996 |
| OCSERCA2 *(SERCA2)* | M33834.1 | CGTTGCCTGGTGAATTTCAAC | ATTGCTCAATCACAAGTTCCAGC | polymorph | vanHaeringen et al. 1996 |
| Sat2 *(CSN1S1)* | M77195 | GCTCTCCTTTGGCATACTCC | GCTTTGGATAGGCCCAGATC | polymorph | Mougel et al. 1997 |
| Sat3 *(PLP2/PMP2)* | J03744 | GGAGAGTGAATCAGTGGGTG | GAGGGAAAGAGAGAGACAGG | polymorph | Mougel et al. 1997 |
| Sat4 | M33582 | GGCCAGTGTCCTTACATTTGG | TGTTGCAGCGAATTGGGG | not polymorph | Mougel et al. 1997 |
| Sat5 | X99887 | GCTTCTGGCTTCAACCTGAC | CTTAGGGTGCAGAATTATAAGAG | polymorph | Mougel et al. 1997 |
| Sat7 | X99888 | GTAACCACCCATGCACACTC | GCACAATACCTGGGATGTAG | polymorph | Mougel et al. 1997 |
| Sat8 | X99889 | CAGACCCGGCAGTTGCAGAG | GGGAGAGAGGGATGGAGGTATG | polymorph | Mougel et al. 1997 |
| Sat12 | X99891 | CTTGAGTTTTAAATTCGGGC | GTTTGGATGCTATCTCAGTCC | polymorph | Mougel et al. 1997 |
| Sat13 | X99892 | CAGTTTTGAAGGACACCTGC | GCCTCTACCTTTGTGGGG | polymorph | Mougel et al. 1997 |
| Sat16 | X99890 | AATCAGCCTCTATGAATTCCC | AATGCTACATGGTAACCAGGC | no fragment | Mougel et al. 1997 |
| Sol33 | X94683 | GAAGGCTCTGAGATCTAGAT | GGGCCAATAGGTACTGATCCATGT | polymorph | Surridge et al., 1997 |
| sol44 | X94684 | GGCCCTAGTCTGACTCTGATTG | GGTGGGGCGGCGGGTCTGAAAC | non-specific fragment | Surridge et al., 1997 |
| Sol51 | X94685 | CTGCATGTAGGGTTTGTGTGT | AACGGAAGAAGACACTATCTCTG | polymorph | Surridge et al., 1997 |
| sol62 | X94686 | TGCCTTTAGGATTGGTCTATCTCTG | GCGGGAGAGGGGGAGAGGGGGAGAG | no fragment | Surridge et al., 1997 |
| sol74 | X94687 | AATGGCTTAGTGCTAAACTAGAC | GCTCGGTACCCCCTCATGTTTG | no fragment | Surridge et al., 1997 |

The references cited here are listed below:

1. Korstanje R, Gillissen GF, Kodde LP, Den Bieman M, Lankhorst A, Van Zutphen LF, Van Lith HA: **Mapping of microsatellite loci and association of aorta atherosclerosis with LG VI markers in the rabbit**. *Physiological genomics* 2001a, **6**(1):11-18.
2. Korstanje R, Gillissen GF, den Bieman MG, Versteeg SA, van Oost B, Fox RR, van Lith HA, van Zutphen LF: **Mapping of rabbit chromosome 1 markers generated from a microsatellite-enriched chromosome-specific library**. *Animal genetics* 2001b, **32**(5):308-312.
3. Korstanje R, Gillissen GF, Versteeg SA, van Oost BA, Bosma AA, Rogel-Gaillard C, van Zutphen LF, van Lith HA: **Mapping of rabbit microsatellite markers using chromosome-specific libraries**. *The Journal of heredity* 2003, **94**(2):161-169.
4. Ferrand N, Azevedo M, Mougel F: **A diallelic short tandem repeat (CCCCG)4 or 5, located in intron 1 of rabbit alpha-globin gene**. *Animal genetics* 2000, **31**(1):74-75.
5. Chantry-Darmon C, Urien C, Hayes H, Bertaud M, Chadi-Taourit S, Chardon P, Vaiman D, Rogel-Gaillard C: **Construction of a cytogenetically anchored microsatellite map in rabbit**. *Mammalian genome : official journal of the International Mammalian Genome Society* 2005, **16**(6):442-459.
6. Rico C, Rico I, Webb N, Smith S, Bell D, Hewitt G: **Four polymorphic microsatellite loci for the European wild rabbit, Oryctolagus cuniculus**. *Animal genetics* 1994, **25**(5):367.
7. Surridge AK, Bell DJ, Rico C, Hewitt GM: **Polymorphic microsatellite loci in the European rabbit (*Oryctolagus cuniculus*) are also amplified in other lagomorph species**. *Animal genetics* 1997, **28**(4):302-305.
8. van Haeringen WA, den Bieman M, van Zutphen LF, van Lith HA: **Polymorphic microsatellite DNA markers in the rabbit (Oryctolagus cuniculus)**. *Journal of experimental animal science* 1996, **38**(2):49-57.
