# Supplementary material for: A comprehensive linkage map and QTL map for carcass traits in a cross between Giant Grey and New Zealand White rabbits
Source: BMC Genet. 2015 Feb 11;16:16. doi: 10.1186/s12863-015-0168-1 (PMC4330979; doi:10.1186/s12863-015-0168-1)
Supplement: Additional file 2: Table S2. — Summary of the information for markers that were integrated into the genetic map [1-4,6-8,10,12-17]. [file 12863_2015_168_MOESM2_ESM.pdf]

Additional file 2

Table S2: Summary of the information for markers that were integrated into the genetic map

OCU/ LG	Marker symbol[1] *(associated gene)*	Accession number	No[2]	Chromosomal positions: Cytogenetic band [Reference]	Chromosomal positions: Physical Chr.: Mb (start)[3]	Genetic position (Kosambi cM): Berlin Map Average	Berlin Map Female	Berlin Map Male	INRA Map	Utrecht Map	Het. F_1[4]	IM[5]
OCU1												
LG1	D1L1B10	AF389352	3	nd[6] [2]	1:11.669725	0.0	0.0	0.0			0.52	392
LG1	Sol51	X94685	3	nd[6] [10]	1:18.934007	5.8	9.6	4.3			0.62	585
LG1	**D1Utr2**	AF389367	3	1 [2]	1:23.922373	7.0	15.1	4.3		12.9	0.59	345
LG1	D1L2B4	AF389358	3	nd[6] [2]	1:31.864989	14.4	23.2	12.9			0.52	246
LG1	D1Utr7	AF389355	5	1 [2]	1:46.450792	21.4	34.6	12.9		24.6	0.83	705
LG1	INRACCDDV0236 *(TJP2)*	AJ874569	2	1p21.3-p21.1[4, 12]	1:55.571101	27.1	43.2	15.4			0.31	177
LG1	**INRACCDDV0269** *(TJP2)*	AJ874595	2	1p21.3-p21.1 [1, 4, 12]	1:55.598578	27.1	43.2	15.4	43.4		0.31	178
LG1	**Sat13**	X99892	4	1p12 [2, 7, 8, 13]	1:63.440888	31.7	50.0	17.9		27.2	0.52	422
LG1	INRACCDDV0345 *(PSAT1)*	AJ874661	3	1p12 [4, 12]	1:64.769638	32.7	51.5	17.9			0.62	344
LG1	INRACCDDV0240 *(DAPK1)*	AJ874573	2	1p11dist [4, 12]	1:73.481778	36.8	56.7	21.0			0.48	249
LG1	INRACCDDV0299	AJ874621	2	nd[6] [4]	1:75.221585	37.4	57.7	22.3			0.72	464
LG1	**D1Utr3**	AF389359	2	1 [2]	UN0044:236839	39.9	61.0	23.7		35.0	0.80	655
LG1	D1L7C11	AF389369	2	nd[6] [2]	1:101.865544	53.1	69.9	42.1			0.83	527
LG1	**INRACCDDV0271** *(HTR3B)*	AJ874597	2	1p11dist/ 1q14 [1, 4, 12]	1:102.427689	53.1	69.9	42.1	99.0		0.53	331
LG1	INRACCDDV0252 *(HTR3B)*	AJ874583	4	1q14 [4, 12]	1:102.503351	53.1	69.9	42.1			0.55	267
LG1	**INRACCDDV0320** *(SLN)*	AJ874640	4	1q14 [1, 4, 12]	1:108.750414	56.8	74.5	44.7	111.2		0.74	655
LG1	D1L8C9	AF389374	2	nd[6] [2]	1:109.935529	57.4	75.4	45.1			0.66	426
LG1	OCPRG5 *(PRG5)*	M14547.1	3	nd[6] [6]	1:115.601359	61.1	81.8	45.6			0.66	413
LG1	INRACCDDV0136	AJ874476	2	nd[6] [4]	1:129.254095	70.5	95.5	51.6			0.61	517
LG1	**D1Utr4**	AF389353	2	1 [2]	1:140.830836	74.4	103.2	53.9	152.4	67.3	0.41	399
LG1	INRACCDDV0302	AJ874624	3	nd[6] [4]	UN0035:2.586312	88.8	115.4	68.2			0.66	491
LG1	**INRACCDDV0169**	AJ874508	3	1q21.5 [1, 4]	1:158.578780	90.4	116.9	69.9	176.5		0.84	654
LG1	**D1Utr5**	AF389357	2	1 [2]	1:167.752362	96.0	124.7	73.0		86.9	0.28	402
LG1	**D1Utr6**	AF389354	2	1q24-q26 [2, 13]	1:185.813547	110.6	153.2	75.7		97.0	0.84	581
LG1	**INRACCDDV0298**	AJ874620	2	1q27dist [1, 4]	UN0303:119096	126.8	160.1	101.1	nl[7]		0.97	660
OCU2												
LG2	**INRACCDDV0192**	AJ874530	3	2p21.3dist [1, 4]	2:29.010004	0.0	0.0	0.0	nl[7]		0.83	522
LG2	**INRACCDDV0173**	AJ874511	5	2q14-q25 [1, 4]	2:103.450832	41.4	55.4	31.5	nl[7]		0.91	735
LG2	INRACCDDV0077 *(FSHR)*	AJ874421	5	2q22 [4, 12]	2:137.458194	72.7	92.3	49.8			0.50	356
OCU3												
LG3	D6L3H10	AF421925	3	nd[6] [3]	UN0969:35647	0.0	0.0	0			0.66	426
LG3	**INRACCDDV0036** *(CD14)*	AJ874398	3	3p21prox [1, 4, 12]	3:22.734617	26.4	23.3	31.4	0.0		0.95	721
LG3	INRACCDDV0159	AJ874499	3	nd[6] [4]	3:31.608853	34.9	32.7	39.2			0.55	379
LG3	INRACCDDV0110	AJ874452	4	nd[6] [4]	3:51.793955	49.5	50.2	51.2			0.62	549
LG3	INRACCDDV0111	AJ874453	5	nd[6] [4]	3:51.794186	50.1	51.4	51.2			1.00	632
LG3	Sat5	X99887	3	nd[6] [8]	3:52.120053	50.7	52.2	51.6			0.90	543
LG3	**Sol33**	X94683	4	3q11 [3, 10, 13]	3:67.506294	54.6	56.4	55.6		16.0	0.60	657
LG3	INRACCDDV0158	AJ874498	3	nd[6] [4]	3:67.808093	54.6	56.4	55.6			0.69	556
LG3	INRACCDDV0225 *(ASPH)*	AJ874558	3	3q16 [4, 12]	3:89.390826	59.5	65.0	55.6			0.62	424
LG3	INRACCDDV0129	AJ874470	3	nd[6] [4]	3:97.054015	72.0	88.5	61.9			0.28	115
LG3	**Sat3** *(PLP2/PMP2)*	J03744	7	3q14-3q15 [3, 8, 13, 14]	3:97.128709	72.0	88.5	61.9		24.6	0.71	616
LG3	**INRACCDDV0203**	AJ874540	3	3q22-q23 [1, 4]	3:132.701292	90.5	122.0	70.4	nl[7]		0.54	612
OCU4												
LG4a	INRACCDDV0340 *(NCOA6)*	AJ874657	2	4p13 [4, 15]	4:4.997753	0.0	0.0	0.0			0.38	298
LG4a	INRACCDDV0314 *(PRNP)*	AJ874635	3	4p13 [4, 12]	4:11.430617	16.6	19.3	6.8			0.61	386
LG4a	INRACCDDV0333 *(PRNP)*	AJ874650	3	4p13 [4, 12]	4:11.438746	19.7	22.9	9.2			0.78	608
LG4a	**INRACCDDV0022** *(ERBB3)*	AJ874385	2	4q11 [1, 4, 12]	4:39.642327	30.8	34.2	20.0	0.0[8]		0.76	476
LG4a	INRACCDDV0040 *(ERBB3)*	AJ874400	4	4q11 [4, 12]	4:39.692417	31.3	34.2	21.0			0.83	659
LG4a	D6L2H3	AF421920	2	nd[6] [3]	UN0016:1.423853	32.2	35.1	22.0			0.22	331
LG4a	**INRACCDDV0100**	AJ874442	3	4 [1, 4]	4:40.214873	33.9	35.4	27.5	nl[7]		0.31	149
LG4a	**INRACCDDV0182**	AJ874520	4	4q13 [4]	4:47.789199	36.6	38.6	27.5	0.0[9]		0.62	501
OCU4												
LG4b	**INRACCDDV0248** *(PMCH)*	AJ874579	4	4q15.1-q15.2 [1, 4, 12]	4:81.470379	0.0	0.0	0.0	68.4[8]		0.46	230
LG4b	**INRACCDDV0228**	AJ874561	3	4 [1, 4]	UN0068:787523	52.5	50.2	65.3	0.0[9]		0.28	104
LG4b	INRACCDDV0194	AJ874532	3	nd[6] [4]	UN0112:177215	61.5	58.0	75.9			0.66	527
OCU5												
LG5	**INRACCDDV0282**	AJ874606	3	5 [1, 4]	UN0660:37685	0.0	0.0	0.0	0.0		0.65	413
LG5	**D5Utr4**	AF421907	5	5 [3]	UN0091:788280	11.3	8.4	20.1		0.0	0.83	528
LG5	D5L1C3	AF421908	3	nd[6] [3]	UN0024:2.971584	21.6	22.6	20.4			0.50	293
LG5	**INRACCDDV0142**	AJ874482	3	5 [1, 4]	5:3.956473	22.2	23.2	27.2	32.7		0.83	562
LG5	**D5Utr2**	AF421913	5	5 [3]	UN0024:544852	26.0	27.5	27.2		6.7	0.69	458
LG5	INRACCDDV0039 *(LCAT)*	AJ874399	2	5q14 [4, 12]	5:23.468490	54.2	63.8	49.8			0.73	565
LG5	**INRACCDDV0211** *(HAS3)*	AJ874545	4	5q14 [1, 4, 12]	5:24.513058	64.2	74.0	50.2	77.7		0.61	279
OCU6												
LG6	**INRACCDDV0290**	AJ874613	2	6p14prox [1, 4]	6:4.700318	0.0	0.0	0.0	0.0		0.55	467
LG6	INRACCDDV0287	AJ874610	4	nd[6] [4]	6:7.089687	4.7	9.1	0.9			0.70	507
LG6	**D6Utr2**	AF421922	3	6 [3]	6:9.765056	14.9	19.5	11.8		42.6	0.19	91
LG6	D6L2F1	AF421919	3	nd[6] [3]	6:16.072792	23.4	31.0	16.1			0.53	411
LG6	**INRACCDDV0214** *(EIF3S8)*	AJ874548	3	6p12prox [1, 4, 12]	6:18.674086	27.8	34.2	21.6	34.2		0.76	444
LG6	D6L2B5	AF421918	3	nd[6] [3]	UN0054:856279	29.1	37.9	21.6			0.71	513
LG6	INRACCDDV0120	AJ874462	3	6q12prox [4]	6:26.244933	30.5	38.4	22.8			0.41	357
LG6	**D6Utr4**	AF421916	5	6 [3]	6:25.038137	31.6	40.2	23.6	26.4	16.6	0.68	512

Table S2: continued

OCU/ LG	Marker symbol[1] *(associated gene)*	Accession number	No[2]	Chromosomal positions: Cytogenetic band [Reference]	Chromosomal positions: Physical Chr.: Mb (start)[3]	Genetic position (Kosambi cM): Berlin Map Average	Berlin Map Female	Berlin Map Male	INRA Map	Utrecht Map	Het. F_1[4]	IM[5]
OCU7												
LG7	**D7Utr6** *(PODXL)*		3	7 [3]	7:10.098532	0.0	0.0	0.0		23.9	0.55	406
LG7	INRACCDDV0311 *(CALU)*	AJ874633	2	7p21prox [4, 15]	7:15.768489	2.9	2.6	3.9			0.66	312
LG7	INRACCDDV0231 *(GPR37)*	AJ874564	2	7p21-p12 [4, 12]	7:19.767546	5.2	4.1	7.9			0.62	312
LG7	**INRACCDDV0221** *(GPR37)*	AJ874555	3	7p21-p12 [1, 4, 12]	7:19.825055	8.9	10.2	7.9	nl[7]		0.57	370
LG7	Sat12	X99891	4	nd[6] [8]	7:30.647593	17.7	18.7	13.5			0.68	539
LG7	INRACCDDV0093	AJ874436	3	nd[6] [4]	7:41.036817	22.7	29.6	15.2			0.83	510
LG7	D7L2F2	AF421933	4	nd[6] [3]	7:60.680119	29.6	37.5	21.1			0.60	585
LG7	INRACCDDV0323 *(PROC)*	AJ874642	5	7q14 [4, 15]	7:59.306464	30.5	37.9	22.4			1.00	630
LG7	INRACCDDV0336 *(PROC)*	AJ874653	3	7q14 [4, 15]	7:59.289400	33.7	42.7	24.0			0.83	504
LG7	**INRACCDDV0163**	AJ874502	4	7q21dist [1, 4]	7:117.531438	68.0	91.4	48.3	nl[7]		0.81	618
LG7	INRACCDDV0164	AJ874503	3	nd[6] [4]	7:117.531629	69.3	93.0	48.7			0.72	473
LG7	**D7Utr3**	AF421935	3	7 [3]	7:118.659538	70.9	93.0	55.1		87.9	0.28	123
LG7	MSTN-SNP (c.-125T>C)	NM_001109821	2	nd[6]	7:130.429151	75.9	99.8	55.8			0.36	240
LG7	MSTN-SNP (c.373+234G>A)	NM_001109821	2	nd[6]	7:130.429151	75.9	99.8	55.8			0.56	523
LG7	MSTN-SNP (c.747+34C>T)	NM_001109821	2	nd[6]	7:130.429151	75.9	99.8	55.8			0.36	197
LG7	**D7Utr4**	AF421932	2	7 [3]	7:151.015154	88.1	115.3	63.8		102.2	0.74	526
LG7	D7L1B10	AF421926	3	nd6 [3]	7:157.320400	90.8	118.5	66.2			1.00	665
LG7	INRACCDDV0092	AJ874435	3	7q25 [4]	7:157.491661	92.4	119.9	67.9			1.00	614
LG7	**D7Utr5**	AF421930	3	7 [3]	7:161.912736	96.2	124.0	71.6		108.6	0.89	678
LG7	D12L1H3	AF421943	2	nd[6] [3]	7:167.212085	98.1	125.0	74.0			0.74	564
OCU8												
LG8	**INRACCDDV0074**	AJ874418	3	8 [1, 4]	8:37.469977	0.0	0.0	0.0	20.1		0.14	54
LG8	INRACCDDV0080 *(SLC6A12)*	AJ874423	3	8p12 [4, 15]	8:37.478525	0.0	0.0	0.0			0.40	245
LG8	**INRACCDDV0087** *(SLC6A12)*	AJ874430	5	8p12 [1, 4, 15]	8:37.475734	0.7	0.0	1.2	52.1		0.66	388
LG8	INRACCDDV0165	AJ874504	3	nd[6] [4]	UN0007:2.539579	6.1	6.5	5.9			0.79	632
LG8	**INRACCDDV0341** *(TPT1)*	AJ874658	4	8q13.3-q21 [1, 4, 15]	8:56.020048	19.0	25.8	13.7	80.1		0.62	360
LG8	**INRACCDDV0021** *(SLC15A1)*	AJ874384	4	8q24 [1, 4, 12]	8:100.476949	49.6	78.9	34.5	119.7		0.71	579
OCU9												
LG9	**INRACCDDV0184**	AJ874522	3	9 [1, 4]	UN0005:3.059813	0.0	0.0	0.0	0.0		0.22	102
LG9	D0Utr16/ D12L1C2	AF421939	4	nd[6] [3]	UN0005:220525	10.0	0.0	18.3			0.90	737
LG9	INRACCDDV0005 *(GPX1)*	AJ874371	4	9p13 [4, 12]	9:16.925406	33.3	28.8	36.4			0.86	610
LG9	**INRACCDDV0274** *(ITIH3)*	AJ874600	2	9p13prox [1, 4, 12]	9:19.829246	35.5	31.4	38.1	32.8		0.83	587
LG9	INRACCDDV0296	AJ874618	3	nd[6] [4]	9:29.041225	44.6	42.2	45.8			0.55	525
LG9	INRACCDDV0016 *(NPC1)*	AJ874380	4	9q13 [4, 12]	9:64.721594	60.2	60.9	58.7			0.59	525
LG9	**INRACCDDV0010**	AJ874375	4	9q13 [1, 4, 12]	9:64.784532	60.2	60.9	58.7	87.0		0.83	658
LG9	**INRACCDDV0146**	AJ874486	3	9 [1, 4]	9:66.056608	61.5	62.1	60.1	49.6		0.81	596
LG9	INRACCDDV0344 *(MAPRE2)*	AJ874660	3	9q14.2 [4, 15]	9:76.418691	73.1	75.2	63.6			0.55	253
LG9	**INRACCDDV0155**	AJ874495	3	9q15.1 [1, 4]	9:87.333291	87.9	88.4	83.0	119.5		0.48	254
LG9	**INRACCDDV0017** *(CYB5)*	AJ874381	2	9q17 [1, 4, 14]	9:114.418655	102.4	97.4	104.5	194.7		0.13	92
OCU10												
LG10	INRACCDDV0004 *(CALR)*	AJ874370	4	10p12 [4, 14]	UN0287:20813	0.0	0.0	0.0			0.26	116
LG10	INRACCDDV0006 *(ICAM5)*	AJ874372	3	10p12 [4, 12]	UN0135:129426	13.5	14.9	11.1			0.90	555
LG10	INRACCDDV0145	AJ874485	3	nd[6] [4]	10:13.560088	31.4	38.0	24.3			0.97	672
LG10	Sat7	X99888	3	nd[6] [8]	10:21.599399	41.7	51.0	32.4			0.52	374
LG10	INRACCDDV0025 *(STK17A)*	AJ874388	3	10q15 [4, 12]	10:26.306649	44.6	54.1	34.4			1.00	738
LG10	INRACCDDV0076 *(DLX5)*	AJ874420	3	10q15 [4, 12]	10:32.224844	50.1	58.2	42.6			0.66	418
LG10	D10Utr1 *(WAP)*	NM_001082390	3	10q16 [6, 16]	10:45.322775	65.6	70.5	60.2			0.60	555
LG10	**INRACCDDV0304** *(EGFR)*	AJ874626	3	10q16ter [1, 4, 15]	10:45.727001	74.6	73.8	71.9	nl[7]		0.60	569
OCU11												
LG11	**INRACCDDV0183**	AJ874521	3	11p11.1-p11.2 [1, 4]	11:27.133319	0.0	0.0	0.0	0.0		0.31	178
LG11	INRACCDDV0108	AJ874450	4	11q13prox [4]	11:52.391639	10.2	20.4	0.5	nl[7]		0.95	754
LG11	**INRACCDDV0237** *(NNT)*	AJ874570	5	11q13-q14 [1, 4, 12]	11:64.364077	19.6	33.5	6.2	66.0		0.97	773
OCU12												
LG12	D12L4A1	AF421945	3	nd[6] [3]	UN0049:776653	0.0	0.0	0.0			0.80	635
LG12	D12L1E11	AF421941	4	nd[6] [3]	UN0017:1.450742	9.9	5.6	14.7			0.93	701
LG12	INRACCDDV0191	AJ874529	2	nd[6] [4]	12:12.395105	25.9	21.8	30.0			0.86	522
LG12	**D12Utr1** *(RLADPA1)*	M22640	4	12q11 [3, 7, 17]	12:23.542148	29.6	24.5	35.1		0.0	0.89	559
LG12	OCRLADF4 *(RLADF4)*	X60986	2	nd[6] [6]	UN0072:11751	32.0	24.6	38.6			0.54	546
LG12	**INRACCDDV0201**	AJ874538	2	12q16 [1, 4]	12:77.126750	64.6	84.1	59.2	0.0		0.55	595
LG12	**INRACCDDV0176**	AJ874514	4	12q23dist [1, 4]	12:128.433337	94.5	124.2	77.4	nl[7]		0.20	114
OCU13												
LG13	INRACCDDV0198	AJ874536	2	nd[6] [4]	13:4.371301	0.0	0.0	0.0			0.76	626
LG13	INRACCDDV0230	AJ874563	4	14p11dist [4]	13:10.557452	8.7	8.4	8.7			0.71	499
LG13	INRACCDDV0219 *(MYH11)*	AJ874553	4	6p12-p13 [4, 12]	13:10.596261	8.7	8.4	8.7			0.69	507
LG13	**INRACCDDV0106**	AJ874448	2	13 [1, 4]	13:12.692600	12.4	13.7	11.2	0.0		0.33	170
LG13	OCELAMB *(ELAMB)*	M91004	2	nd[6] [6]	13:21.851839	15.0	13.7	16.4			0.83	645
LG13	**INRACCDDV0297**	AJ874619	3	13q21prox [1, 4]	13:26.717719	16.3	14.8	16.4	48.6		0.34	152
LG13	OCCRP *(CRP)*	M14538.1	3	nd[6] [6]	13:33.404947	23.4	20.5	26.9			0.38	260
LG13	INRACCDDV0293	AJ874616	8	nd[6] [4]	13:33.420599	26.6	25.8	26.9			1.00	649
LG13	INRACCDDV0027 *(CD1B)*	AJ874390	3	13q21 [4, 12]	13:34.788232	27.9	27.1	28.2			0.36	332
LG13	INRACCDDV0153	AJ874493	4	nd[6] [4]	13:42.013148	35.3	33.8	36.2			0.61	347
LG13	**INRACCDDV0139**	AJ874479	3	13 [1, 4]	13:47.564061	38.3	37.4	38.7	84.6		0.45	392
LG13	INRACCDDV0270 *(BCAS2)*	AJ874596	2	13q22-q23 [4, 12]	13:49.682570	40.0	42.2	38.7			0.67	429
LG13	**INRACCDDV0177**	AJ874515	2	13 [1, 4]	13:57.547003	47.9	49.8	45.9	102.8		0.50	367
LG13	**INRACCDDV0289**	AJ874612	3	13 [1, 4]	13:111.882614	76.4	91.4	64.6	214.8		0.93	506
LG13	**INRACCDDV0014** *(SLC2A1)*	AJ874378	2	13q31-q32 [1, 4, 14]	13:123.935975	84.9	99.3	74.2	181.5		0.62	389
LG13	**INRACCDDV0151**	AJ874491	2	13 [1, 4]	UN0006:4.131875	91.7	107.5	79.2	180.5		0.48	324
LG13	**INRACCDDV0291**	AJ874614	2	13 [1, 4]	UN0006:5.102365	95.0	110.0	83.7	243.3		0.43	343
LG13	INRACCDDV0310 *(PTAFR)*	AJ874632	4	13q33 [4, 15]	13:137.381150	99.2	115.2	86.2			1.00	755

Table S2: continued

OCU/ LG	Marker symbol[1] *(associated gene)*	Accession number	No[2]	Chromosomal positions		Genetic position (Kosambi cM)					Het. F_1[4]	IM[5]
				Cytogenetic band [Reference]	Physical Chr.: Mb (start)[3]	Berlin Map Average	Berlin Map Female	Berlin Map Male	INRA Map	Utrecht Map		
OCU14												
LG14	**INRACCDDV0337** *(SIAH2)*	AJ874654	3	14q13prox [1, 4, 15]	14:44.599073	0.0	0.0	0.0	34.9		0.57	281
LG14	**INRACCDDV0313** *(HES1)*	AJ874634	4	14q21prox [1, 4, 12]	14:89.981493	37.4	40.6	28.1	nl[7]		0.97	755
LG14	**INRACCDDV0140**	AJ874480	3	14q23 [1, 4]	14:121.357640	50.8	55.7	40.5	70.3		0.58	580
LG14	**INRACCDDV0241** *(TIAM1)*	AJ874574	4	14q25 [1, 4, 12]	14:161.237466	79.6	90.5	54.2	92.4		0.67	442
LG14	**INRACCDDV0162**	AJ874501	4	14 [1, 4]	UN0209:452477	86.6	98.4	60.0	108.2		0.64	433
OCU15												
LG15	INRACCDDV0103	AJ874445	3	nd[6] [4]	UN0003:7.861545	0.0	0.0	0.0			0.66	496
LG15	**INRACCDDV0286**	AJ874609	3	15q11.3 [1, 4]	15:18.057583	14.9	18.8	7.4	47.6		0.45	337
LG15	**INRACCDDV0125**	AJ874466	4	15 [1, 4]	15:20.804619	17.3	20.7	10.8	24.9		1.00	775
LG15	**INRACCDDV0143**	AJ874483	2	15q12/ 15q21prox [1, 4]	15:108.340756	23.3	30.2	13.0	nl[7]		0.83	508
LG15	INRACCDDV0091	AJ874434	2	nd[6] [4]	15:35.956631	37.7	51.7	22.0			0.52	477
LG15	**INRACCDDV0288**	AJ874611	3	15 [1, 4]	15:53.373658	46.9	60.6	30.8	64.7		0.84	747
LG15	INRACCDDV0115	AJ874457	3	nd[6] [4]	15:60.785483	51.9	69.1	32.1			0.93	584
LG15	INRACCDDV0044 (*ALB*)	AJ874402	2	15q23 [4, 12]	15:76.663725	62.2	79.6	42.5			0.38	367
LG15	INRACCDDV0306 (*GC*)	AJ874628	2	15q23dist [4, 15]	15:78.231304	62.2	79.6	42.5			0.86	494
LG15	**INRACCDDV0303** *(SLC4A4)*	AJ874625	3	15q23dist [1, 4, 15]	15:78.678247	63.4	81.0	42.5	136.1		0.48	223
LG15	Sat2 *(CSN1S1)*	M77195	3	nd[6] [8]	15:79.905947	63.4	81.0	42.5			0.45	226
LG15	OCAS1CG *(CSN1S1)*	AY284844.1	4	nd[6] [6]	15:79.905947	63.4	81.0	42.5			0.55	348
LG15	INRACCDDV0018 *(CSN1S1)*	AJ874382	2	15q23 [4, 14]	15:79.939447	64.3	81.0	45.0			0.61	318
LG15	**INRACCDDV0294**	AJ874617	3	15 [1, 4]	UN0015:2.339043	78.7	91.7	63.3	90.0		1.00	730
OCU16												
LG16	**INRACCDDV0148**	AJ874488	2	16p12.1 [1, 4]	16:2.262135	0.0	0.0	0.0	0.0		0.28	175
LG16	INRACCDDV0279	AJ874604	4	nd[6] [4]	16:23.048782	29.4	37.2	18.0			0.86	428
LG16	**INRACCDDV0185**	AJ874523	2	16q23 [1, 4]	16:82.956921	91.2	90.6	118.0	74.4		0.48	315
OCU17												
LG17	**INRACCDDV0172**	AJ874510	3	17q21dist [1, 4]	17:55.188981	0.0	0.0	0.0	33.2		0.53	307
LG17	INRACCDDV0217 *(GMFB)*	AJ874551	4	17q23prox [4, 15]	17:73.200829	10.6	14.3	7.0			0.93	649
LG17	Sat8	X99889	3	nd[6] [8]	17:78.000869	15.1	14.8	13.4			0.31	292
OCU18												
LG18	**INRACCDDV0280**	AJ874605	3	18q12prox [1, 4]	18:3.817803	0.0	0.0	0.0	nl[7]		0.73	677
LG18	INRACCDDV0218 *(ARFGEF1)*	AJ874552	4	3q14 [4, 12]	18:68.515123	8.7	8.3	9.2			1.00	577
LG18	**INRACCDDV0123**	AJ874464	2	18 [1, 4]	18:9.868534	19.0	19.8	18.4	8.1[10]		0.73	633
LG18	**INRACCDDV0188**	AJ874526	3	18 [1, 4]	18:23.594801	36.7	39.9	34.5	0.0[11]		0.44	440
LG18	INRACCDDV0258 *(MINPP1)*	AJ874588	2	18q23 [4, 12]	18:35.388785	43.3	53.9	34.6			0.17	198
LG18	INRACCDDV0029 *(CYP2C18)*	AJ874392	4	18q31 [4, 12]	18:42.614826	48.5	56.1	40.0			1.00	600
LG18	**INRACCDDV0023** *(CYP2C18)*	AJ874386	3	18q31 [1, 4, 12]	18:42.669764	48.5	56.1	40.0	53.9[10]		1.00	600
LG18	INRACCDDV0063 *(CYP2C18)*	AJ874409	4	18q31 [4, 12]	18:42.699522	48.5	56.1	40.0			1.00	600
LG18	D0Utr10 (*CYP2C4*)	M74203	3	18q24 [6, 13]	18:43.242464	48.6	56.1	40.3			0.92	726
LG18	INRACCDDV0256 *(MSN)*	AJ874586	5	Xq12prox [4, 12]	18:52.168480	53.4	61.9	44.0			1.00	744
LG18	**INRACCDDV0168**	AJ874507	2	18 [1, 4]	18:59.695227	57.7	64.4	50.0	47.0[10]		0.74	637
OCU19												
LG19	**INRACCDDV0234** *(NDEL1)*	AJ874567	3	19q12.3 [1, 4, 12]	19:10.884688	0.0	0.0	0.0	0.0		0.51	523
LG19	D19L1E12	AF421950	2	nd[6] [3]	19:23.208880	19.7	23.5	17.6			0.83	704
LG19	**INRACCDDV0102**	AJ874444	4	19 [1, 4]	19:24.412844	21.8	24.9	20.3	25.2		0.85	731
LG19	**INRACCDDV0094**	AJ874437	4	19q21prox [1, 4]	19:36.155753	30.9	38.5	24.7	36.3		0.54	283
LG19	**D19Utr3**	AF421951	3	19 [3]	19:37.973556	34.6	43.7	26.9		42.0	0.38	164
LG19	**INRACCDDV0071** *(KRT12)*	AJ874415	3	19q21 [1, 4, 12]	19:41.957794	36.9	45.5	30.3	45.9		0.80	670
LG19	**INRACCDDV0193**	AJ874531	5	19 [1, 4]	19:55.797718	54.2	65.0	44.7	60.4		0.78	557
LG19	**D19Utr4**	AF421949	3	19 [3]	UN	67.8	73.5	64.5		84.5	0.93	724
OCU21												
nd[6]	OCERSCA2 *(SERCA2)*	M33834.1	2	nd[6] [6]	21:6.613853	-	-	-			0.31	177
OCUX												
LGX	**INRACCDDV0213** *(PRKCB1)*	AJ874547	6	6p12prox [1, 4, 12]	X:9.618638	-	0.0	-	7.7[12]		0.68	498
LGX	**INRACCDDV0127**	AJ874468	2	6 [1, 4]	X:24.702536	-	18.1	-	45.9[12]		0.95	594
LGX	INRACCDDV0126	AJ874467	2	nd[6] [4]	X:24.702290	-	19.6	-			0.91	602
LGX	DXUtr1	AF389361	6	X [2]	UN0085:798734	-	119.6	-			0.48	750
LGX	INRACCDDV0084 *(TGFB3)*	AJ874427	2	20q12 [4, 14]	X:108.830383	-	139.7	-			0.31	492
nd[6]	INRACCDDV0035 (*CSN3*)	AJ874397	3	15q23dist [4, 12]	15:79.715129	-	-	-			nd[5]	nd[5]
nd[6]	INRACCDDV0157	AJ874497	3	8p11 [4]	8:45.058626	-	-	-			nd[5]	nd[5]
nd[6]	INRACCDDV0204	AJ874541	3	nd[6] [4]	1:77.245221	-	-	-			nd[5]	nd[5]

[1] bold markers previously genetically mapped; [2] number of observed alleles; [3] physical position in Enseml database 73, OryCun2.0 (http://www.ensembl.org/Oryctolagus_cuniculus); [4] heterozygosity in F_1; [5] informative meioses; [6] nd-not determined; [7] nl- not linked; [8] marker assigned to LG4b; [9] marker assigned to LG4a; [10] marker assigned to LG18b; [11] marker assigned to LG 18a; [12] marker assigned to LG6b
