# Supplementary material for: A comprehensive linkage map and QTL map for carcass traits in a cross between Giant Grey and New Zealand White rabbits
Source: BMC Genet. 2015 Feb 11;16:16. doi: 10.1186/s12863-015-0168-1 (PMC4330979; doi:10.1186/s12863-015-0168-1)
Supplement: Additional file 3: Figure S1. — Cytogenetic associated linkage map for the F2 cross GG x NZW. The cytogenetic map (left) of every chromosome is connected to the sex averaged pedigree-specific linkage map (right). The numbers on the right hand side of the linkage maps give the estimated distances between loci in cM (Kosambi), the statistical support for the pair-wise order of markers is given on the left hand side. The total genetic length of every linkage group is given at the bottom of each bar. Connecting lines indicate the cytogenetic positions of microsatellites previously mapped by fluorescence in situ hybridization [1-4,7,12-15,17]. [file 12863_2015_168_MOESM3_ESM.pdf]

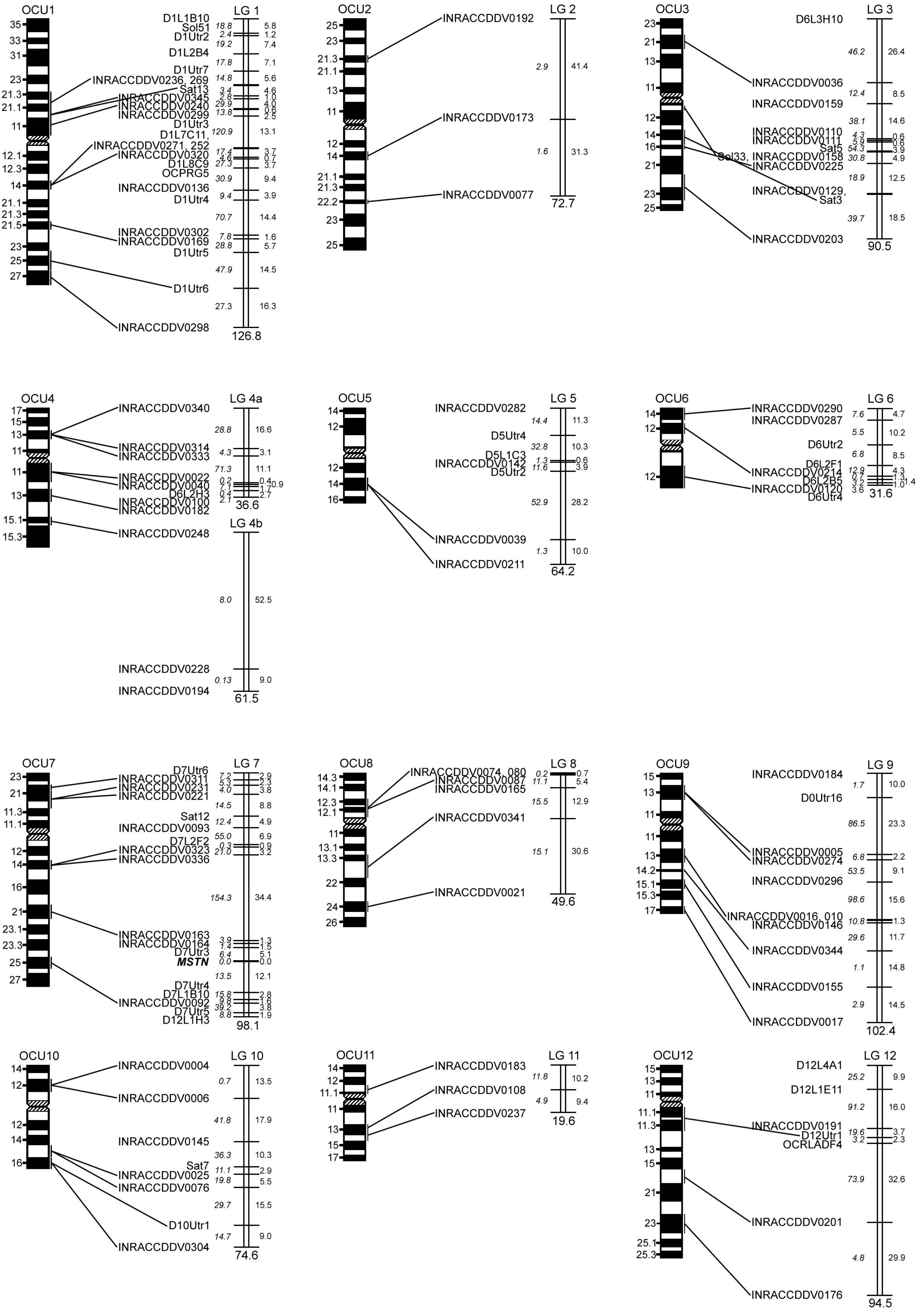

Figure S1: Cytogenetic associated linkage map for the F2 cross GG x NZW. The cytogenetic map (left) of every chromosome is connected to the sex averaged pedigree-specific linkage map (right). The numbers on the right hand side of the linkage maps give the estimated distances between loci in cM (Kosambi), the statistical support for the pair-wise order of markers is given on the left hand side. The total genetic length of every linkage group is given at the bottom of each bar. Connecting lines indicate the cytogenetic positions of microsatellites previously mapped by fluorescence in situ hybridization ([1-4,7,12-15,17].

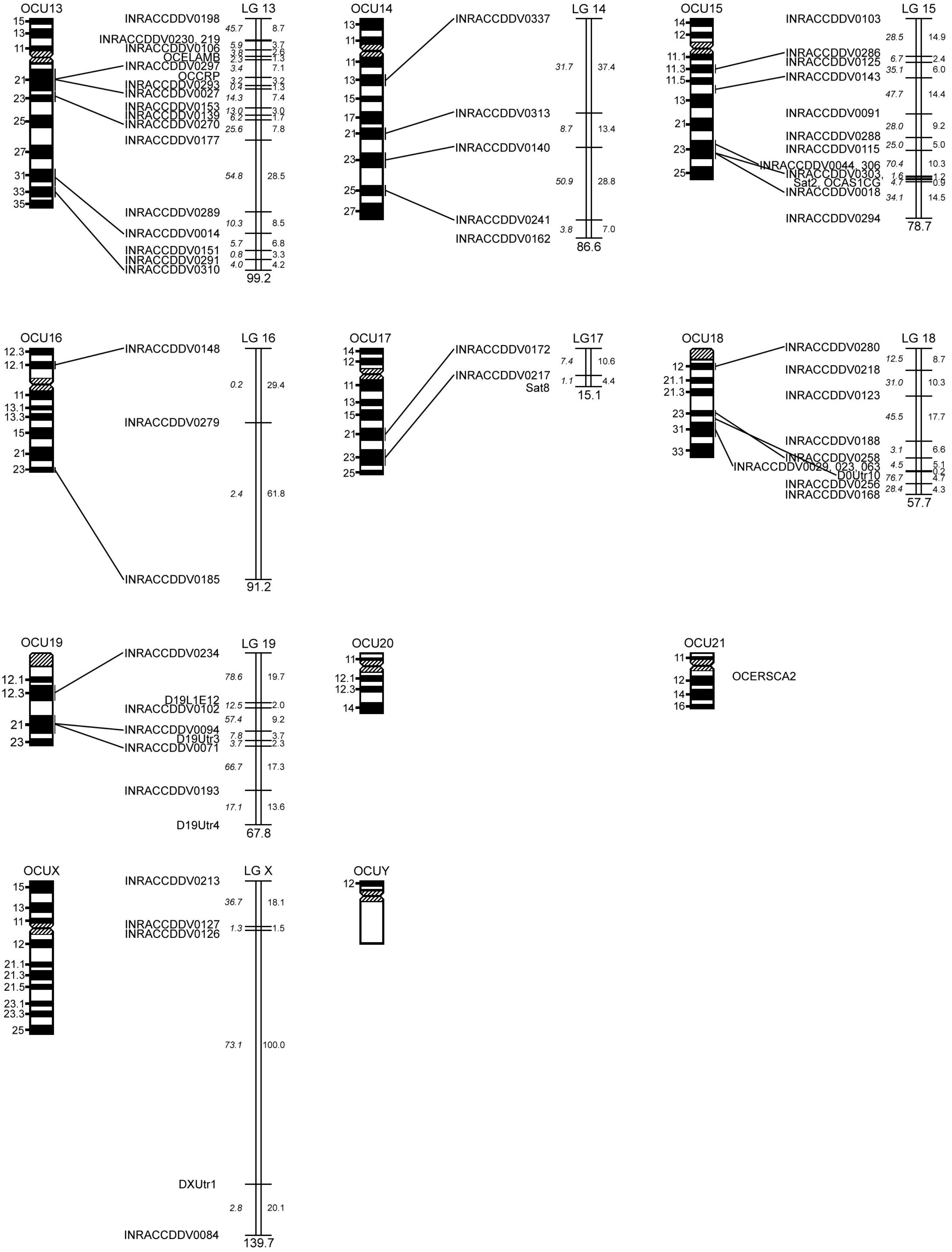

Figure S1: continued
