# Supplementary material for: A comprehensive linkage map and QTL map for carcass traits in a cross between Giant Grey and New Zealand White rabbits
Source: BMC Genet. 2015 Feb 11;16:16. doi: 10.1186/s12863-015-0168-1 (PMC4330979; doi:10.1186/s12863-015-0168-1)
Supplement: Additional file 4: Table S3. — Positions and effects of suggestive QTL for carcass traits of the cross between GG and NZW rabbits. [file 12863_2015_168_MOESM4_ESM.docx]

## Additional file 4

## Table S3: Positions and effects of suggestive QTL for carcass traits of the cross between Giant Grey and New Zealand White rabbits

| OCU/ | | Trait | Model^1^ | cM^2^ | (Mb) | **Flanking markers^3^** | | 95% CI^4^ | | F‑value^5^ | a (SE)^6^ | | d (SE)^7^ | | VF_2_%^8^ |
| --- | --- | --- | --- | --- | --- | --- | --- | --- | --- | --- | --- | --- | --- | --- | --- |
| LG | |  |  |  |  | **left or direct** | right |  | |  |  | |  | |  |
| 1 | Intermediate part weight (g) | | 2 | 36.0 | (71.78) | INRACCDDV0345 | INRACCDDV0240 | 0.0- | 99.5 | 6.61^†^ | **-5.67** | **(1.61)** | -2.25 | (2.28) | 3.98 |
| 1 | Inguinal fat weight (g) | | 1 | 45.0 | (88.12) | D1Utr3 | D1L7C11 | 9.5- | 126.0 | 5.91^†^ | 0.75 | (0.50) | **-2.26** | **(0.75)** | 3.98 |
| 1 | Protein content in *M. long .*(%) | | 1 | 58.0 | (110.85) | D1L8C9 | OCPRG5 | 30.5- | 75.0 | 7.76^†^ | **-0.14** | **(0.06)** | 0.26 | (0.86) | 14.57 |
| 1 | pH_24_ value *M.* *bic. fem.* | | 1 | 94.0 | (164.48) | INRACCDDV0169 | D1Utr5 | 0.0- | 118.5 | 6.16^†^ | -0.01 | (0.01) | **-0.07** | **(0.02)** | 3.72 |
| 2 | Intermediate part weight (g) | | 2 | 0.0 | (29.01) | INRACCDDV0192 |  | 0.0- | 41.0 | 5.82^†^ | **-6.01** | **(1.77)** | 0.89 | (2.53) | 3.52 |
| 2 | Meat weight intermediate part (g) | | 2 | 0.0 | (29.01) | INRACCDDV0192 |  | 0.0- | 72.0 | 4.42^†^ | -3.14 | (1.99) | **9.44** | **(3.88)** | 3.01 |
| 2 | Meat weight hind part (g) | | 2 | 0.0 | (29.01) | INRACCDDV0192 |  | 0.0- | 40.0 | 4.66^†^ | **4.20** | **(1.50)** | 2.57 | (2.15) | 3.17 |
| 2 | Meat colour_24_ L* *M.* *bic. fem.* | | 1 | 42.0 | (104.10) | INRACCDDV0173 | INRACCDDV0077 | 0.0- | 72.0 | 4.39^†^ | **-0.39** | **(0.14)** | 0.23 | (0.21) | 2.88 |
| 3 | Meat weight fore part (g) | | 1 | 60.0 | (89.70) | INRACCDDV0225 | INRACCDDV0129 | 27.0- | 90.0 | 5.42^†^ | 4.15 | (6.41) | **29.32** | **(8.99)** | 3.67 |
| 3 | Hind part weight (g) | | 2 | 85.0 | (122.13) | Sat3 | INRACCDDV0203 | 0.0- | 90.0 | 6.05^†^ | 1.83 | (1.35) | **-7.19** | **(2.23)** | 3.65 |
| 3 | Intermediate part weight (g) | | 2 | 90.0 | (131.74) | Sat3 | INRACCDDV0203 | 6.0- | 90.0 | 5.94^†^ | **-4.93** | **(1.71)** | **5.27** | **(2.64)** | 3.59 |
| 4a | Perirenal fat weight (g) | | 2 | 20.0 | (12.20) | INRACCDDV0333 | INRACCDDV0022 | 18.0- | 34.5 | 4.11^†^ | -0.48 | (0.72) | **2.84** | **(1.03)** | 2.81 |
| 5 | Bone weight intermediate part (g) | | 1 | 11.0 | (nb) | INRACCDDV0282 | D5Utr4 | 0.0- | 61.5 | 4.28^†^ | **1.87** | **(0.64)** | -0.17 | (0.94) | 2.92 |
| 5 | Bone weight hind part (g) | | 1 | 11.0 | (nb) | INRACCDDV0282 | D5Utr4 | 0.0- | 64.0 | 5.02^†^ | **4.40** | **(1.45)** | -2.23 | (2.12) | 3.40 |
| 5 | Scapular fat weight (g) | | 1 | 39.0 | (14.20) | D5Utr2 | INRACCDDV0039 | 21.0- | 61.0 | 5.55^†^ | -1.47 | (0.83) | **4.02** | **(1.48)** | 3.76 |
| 5 | Shear force (N/cm2) | | 1 | 47.0 | (19.08) | D5Utr2 | INRACCDDV0039 | 11.5- | 64.0 | 5.17^†^ | 0.12 | (0.11) | **0.59** | **(0.19)** | 7.47 |
| 7 | Liveweight (g) | | 1 | 91.0 | (157.34) | D7L1B10 | INRACCDDV0092 | 0.0- | 98.0 | 5.45^†^ | **82.66** | **(26.74)** | 45.64 | (39.87) | 3.30 |
| 7 | Scapular fat weight (g) | | 2 | 98.0 | (166.93) | D7Utr5 | D12L1H3 | 25.5- | 98.0 | 7.14^†^ | **-2.27** | **(0.63)** | -1.01 | (0.91) | 4.79 |
| 8 | Hot carcass weight (g) | | 1 | 1.0 | (37.78) | INRACCDDV0087 | INRACCDDV0165 | 0.0- | 49.0 | 4.31^†^ | **34.05** | **(15.97)** | **49.69** | **(24.20)** | 2.63 |
| 8 | Reference carcass weight (g) | | 1 | 1.0 | (37.78) | INRACCDDV0087 | INRACCDDV0165 | 0.0- | 49.0 | 4.13^†^ | **33.09** | **(15.65)** | **46.93** | **(23.71)** | 2.52 |
| 8 | Fore part weight (g) | | 1 | 1.0 | (37.78) | INRACCDDV0087 | INRACCDDV0165 | 0.0- | 49.0 | 4.61^†^ | **13.95** | **(6.60)** | **22.14** | **(10.00)** | 2.81 |
| 8 | Hind part weight (g) | | 1 | 1.0 | (37.78) | INRACCDDV0087 | INRACCDDV0165 | 0.0- | 49.0 | 5.18^†^ | **12.31** | **(5.51)** | **19.60** | **(8.34)** | 3.14 |
| 8 | Liveweight (g) | | 1 | 2.0 | (38.79) | INRACCDDV0087 | INRACCDDV0165 | 0.0- | 46.5 | 4.47^†^ | 53.25 | (28.17) | **100.13** | **(42.91)** | 2.72 |
| 8 | Meat weight hind part (g) | | 1 | 2.0 | (38.79) | INRACCDDV0087 | INRACCDDV0165 | 0.0- | 49.0 | 4.38^†^ | 8.43 | (4.87) | **17.53** | **(7.47)** | 2.98 |
| 8 | Meat weight hind part (g) | | 2 | 19.0 | (56.02) | INRACCDDV0341 |  | 0.0- | 49.0 | 4.16^†^ | **4.62** | **(1.67)** | 2.30 | (3.04) | 2.83 |
| 8 | pH_45_ value *M.* *bic. fem.* | | 1 | 35.0 | (79.27) | INRACCDDV0341 | INRACCDDV0021 | 2.0- | 49.0 | 4.50^†^ | 0.04 | (0.03) | **-0.16** | **(0.06)** | 2.75 |
| 9 | Headweight (g) | | 1 | 53.0 | (48.25) | INRACCDDV0296 | INRACCDDV0016 | 2.5- | 66.0 | 7.22^†^ | 5.83 | (14.53) | **-85.33** | **(22.74)** | 4.32 |
| 9 | Liveweight (g) | | 1 | 60.0 | (64.26) | INRACCDDV0296 | INRACCDDV0016 | 16.0- | 62.0 | 7.34^†^ | **79.45** | **(27.88)** | **-93.65** | **(39.49)** | 4.39 |
| 9 | Hot carcass weight (g) | | 1 | 60.0 | (64.26) | INRACCDDV0296 | INRACCDDV0016 | 0.0- | 72.5 | 5.81^†^ | **40.16** | **(15.73)** | **-46.58** | **(22.30)** | 3.51 |
| 9 | Reference carcass weight (g) | | 1 | 60.0 | (64.26) | INRACCDDV0296 | INRACCDDV0016 | 0.0- | 73.0 | 5.72^†^ | **39.29** | **(15.41)** | **-44.79** | **(21.84)** | 3.46 |
| 9 | Fore part weight (g) | | 1 | 60.0 | (64.26) | INRACCDDV0296 | INRACCDDV0016 | 0.0- | 65.5 | 6.45^†^ | **17.56** | **(6.50)** | **-20.12** | **(9.21)** | 3.89 |
| 9 | Meat weight hind part (g) | | 1 | 60.0 | (64.26) | INRACCDDV0296 | INRACCDDV0016 | 19.5- | 73.0 | 6.04^†^ | **13.63** | **(4.92)** | -12.77 | (6.96) | 4.07 |
| 9 | Hind part weight (g) | | 1 | 61.0 | (65.57) | INRACCDDV0010 | INRACCDDV0046 | 0.0- | 72.0 | 6.96^†^ | **16.11** | **(5.42)** | **-15.92** | **(7.74)** | 4.18 |
| 9 | Bone weight hind part (g) | | 1 | 63.0 | (67.40) | INRACCDDV0046 | INRACCDDV0344 | 10.0- | 88.0 | 5.58^†^ | **4.12** | **(1.47)** | -3.31 | (2.16) | 3.77 |
| 9 | Lipid content of *M. long.* (%) | | 1 | 68.0 | (71.86) | INRACCDDV0046 | INRACCDDV0344 | 8.5- | 77.5 | 5.27^†^ | -0.05 | (0.04) | **0.21** | **(0.07)** | 10.08 |
| 9 | Bone weight fore part (g) | | 2 | 102.0 | (113.67) | INRACCDDV0155 | INRACCDDV0017 | 5.0- | 102.0 | 7.29^†^ | **7.40** | **(1.97)** | -0.58 | (3.77) | 4.88 |
| 9 | Meat colour_24_ b* *M.* *bic. fem.* | | 1 | 102.0 | (113.67) | INRACCDDV0155 | INRACCDDV0017 | 23.0- | 102.0 | 7.17^†^ | -0.16 | (0.16) | **1.04** | **(0.30)** | 4.64 |
| 10 | Bone weight fore part (g) | | 2 | 39.0 | (19.49) | INRACCDDV0145 | Sat7 | 0.0- | 72.5 | 4.84^†^ | -1.60 | (1.29) | **5.76** | **(2.02)** | 3.29 |
| 10 | Intermediate part weight (g) | | 2 | 44.0 | (25.34) | Sat7 | INRACCDDV0025 | 13.0- | 74.0 | 4.74^†^ | 2.70 | (1.51) | **-5.64** | **(2.22)** | 2.89 |
| 10 | Meat weight intermediate part (g) | | 2 | 47.0 | (28.89) | INRACCDDV0025 | INRACCDDV0076 | 0.0- | 74.0 | 6.02^†^ | 2.03 | (1.36) | **-6.38** | **(2.04)** | 4.05 |
| 10 | Meat weight intermediate part (g) | | 1 | 54.0 | (35.52) | INRACCDDV0076 | D10Utr1 | 18.5- | 74.0 | 5.05^†^ | **10.83** | **(3.57)** | -5.19 | (5.86) | 3.42 |
| 11 | Protein content of *M. long.* (%) | | 1 | 2.0 | (32.09) | INRACCDDV0183 | INRACCDDV0108 | 0.0- | 19.0 | 4.41^†^ | 0.03 | (0.06) | **-0.28** | **(0.10)** | 8.83 |
| 11 | Meat colour_45_ b* *M.* *bic. fem.* | | 1 | 4.0 | (37.04) | INRACCDDV0183 | INRACCDDV0108 | 0.0- | 18.0 | 4.71^†^ | -0.21 | (0.12) | **0.46** | **(0.18)** | 3.03 |
| 11 | Perirenal fat weight (g) | | 2 | 12.0 | (54.68) | INRACCDDV0108 | INRACCDDV0237 | 0.0- | 19.0 | 3.97^†^ | **2.09** | **(0.74)** | -0.10 | (1.02) | 2.71 |
| 11 | Bone weight fore part (g) | | 2 | 19.0 | (63.60) | INRACCDDV0108 | INRACCDDV0237 | 0.0- | 19.0 | 4.32^†^ | -2.00 | (1.34) | **-4.62** | **(1.83)** | 2.95 |
| 11 | Meat weight hind part (g) | | 2 | 19.0 | (63.60) | INRACCDDV0108 | INRACCDDV0237 | 0.0- | 19.0 | 5.01^†^ | -2.78 | (1.44) | **4.94** | **(1.97)** | 3.40 |
| 12 | Meat weight fore part (g) | | 2 | 65.0 | (77.81) | INRACCDDV0201 | INRACCDDV0176 | 10.0- | 94.0 | 6.90^†^ | **-7.10** | **(1.92)** | 0.32 | (2.99) | 4.63 |
| 12 | Fore part weight (g) | | 2 | 78.0 | (100.12) | INRACCDDV0201 | INRACCDDV0176 | 10.0- | 94.0 | 6.11^†^ | **-7.38** | **(2.11)** | -1.22 | (4.07) | 3.69 |
| 14 | Bone weight hind part Cg) | | 2 | 0.0 | (44.60) | INRACCDDV0337 |  | 0.0- | 86.0 | 5.06^†^ | **-4.96** | **(1.68)** | -2.89 | (3.28) | 3.42 |
| 14 | Kidney weight (g) | | 1 | 16.0 | (64.01) | INRACCDDV0337 | INRACCDDV0313 | 0.0- | 86.0 | 4.58^†^ | **0.99** | **(0.34)** | 0.05 | (0.67) | 2.78 |
| 15 | Hind part weight (g) | | 2 | 21.0 | (23.55) | INRACCDDV0125 | INRACCDDV0143 | 0.0- | 59.0 | 6.18^†^ | **-4.25** | **(1.24)** | 1.60 | (1.77) | 3.73 |
| 15 | Intermediate part weight (g) | | 2 | 23.0 | (25.04) | INRACCDDV0125 | INRACCDDV0143 | 0.0- | 78.0 | 4.74^†^ | **4.77** | **(1.65)** | -2.58 | (2.32) | 2.88 |
| 15 | Meat weight hind part (g) | | 1 | 50.0 | (57.97) | INRACCDDV0288 | INRACCDDV0115 | 7.0- | 78.0 | 5.07^†^ | 1.55 | (4.90) | **23.27** | **(7.37)** | 3.44 |
| 16 | Headweight (g) | | 1 | 0.0 | (2.26) | INRACCDDV0148 |  | 0.0- | 91.0 | 6.37^†^ | -19.66 | (22.62) | **143.89** | **(41.12)** | 3.83 |
| 16 | Meat colour_24_ a* *M.* *bic. fem.* | | 1 | 9.0 | (8.63) | INRACCDDV0148 | INRACCDDV0279 | 0.0- | 91.0 | 4.54^†^ | 0.09 | (0.30) | **1.80** | **(0.60)** | 2.99 |
| 17 | Intermediate part weight (g) | | 2 | 2.0 | (58.59) | INRACCDDV0172 | INRACCDDV0217 | 0.0- | 15.0 | 5.45^†^ | **4.25** | **(1.77)** | **6.10** | **(2.80)** | 3.30 |
| 17 | Meat colour_45_ L* *M.* *bic. fem.* | | 1 | 15.0 | (77.89) | INRACCDDV0217 | Sat8 | 0.0- | 15.0 | 5.51^†^ | **-0.52** | **(0.16)** | -0.02 | (0.24) | 3.53 |
| 17 | Meat colour_24_ L* *M.* *bic. fem.* | | 1 | 15.0 | (77.89) | INRACCDDV0217 | Sat8 | 0.0- | 15.0 | 3.97^†^ | **-0.37** | **(0.14)** | -0.17 | (0.21) | 2.61 |
| 17 | Kidney weight (g) | | 1 | 15.0 | (77.89) | INRACCDDV0217 | Sat8 | 0.0- | 15.0 | 4.13^†^ | -0.16 | (0.23) | **0.96** | **(0.34)** | 2.52 |
| 18 | Meat weight fore part (g) | | 1 | 13.0 | (7.96) | INRACCDDV0218 | INRACCDDV0123 | 9.0- | 57.0 | 6.31^†^ | **20.48** | **(5.86)** | 4.14 | (9.29) | 4.26 |
| 18 | Fore part weight (g) | | 1 | 14.0 | (8.28) | INRACCDDV0218 | INRACCDDV0123 | 9.0- | 57.0 | 5.39^†^ | **22.04** | **(6.75)** | 2.22 | (10.54) | 3.27 |
| 18 | Liveweight (g) | | 1 | 14.0 | (8.28) | INRACCDDV0218 | INRACCDDV0123 | 9.0- | 57.0 | 4.55^†^ | **86.74** | **(29.12)** | 15.67 | (45.44) | 2.76 |
| 18 | Drip loss (%) | | 1 | 43.0 | (34.85) | INRACCDDV0188 | INRACCDDV0258 | 0.0- | 51.5 | 5.00^†^ | 0.09 | (0.07) | **-0.30** | **(0.10)** | 3.04 |
| 19 | Intermediate part weight (g) | | 2 | 67.0 | nb | INRACCDDV0193 | D19Utr4 | 0.0- | 67.0 | 5.60^†^ | -2.13 | (1.67) | **-7.34** | **(2.45)** | 3.39 |
| X | Bone weight fore part (g) | | 1 | 26.0 | (29.19) | INRACCDDV0126 | DXUtr1 | 3.0- | 139.0 | 4.38^†^ | **-7.37** | **(3.10)** | -6.30 | (3.78) | 2.99 |
| X | Bone weight fore part (g) | | 2 | 70.0 | (60.01) | INRACCDDV0126 | DXUtr1 | 0.0- | 132.0 | 4.70^†^ | -6.09 | (6.23) | **-46.39** | **(16.05)** | 3.20 |
| X | Fore part weight (g) | | 2 | 119.0 | (94.33) | INRACCDDV0126 | DXUtr1 | 2.0- | 132.5 | 5.72^†^ | **7.22** | **(2.37)** | -3.59 | (2.55) | 3.46 |
| X | Intermediate part weight (g) | | 2 | 124.0 | (97.83) | DXUtr1 | INRACCDDV0084 | 10.5- | 139.0 | 4.58^†^ | **-7.25** | **(2.73)** | 4.22 | (3.03) | 2.79 |

^1^Model 1-standard QTL model with covariate birthweight; Model 2-standard QTL model with covariate reference carcass weight, ^2^Chromosomal location is given as pedigree-specific cM position; first marker on each chromosome was set at 0 cM. Estimated physical position between the flanking markers in Mb is given in parentheses; ^3^Flanking markers (left and right) of the QTL peak ^4^CI-confidence interval; ^5^F-value is F-statistic for QTL using standard one QTL model; ^6^a-additive effect; ^7^d-dominance effect; the direction of additive and dominance effects is given as GG-allele effect compared to NZW, bold values indicates significant effects; ^8^phenotypic F_2_ variance (%) explained by the QTL; **highly significant at 1% genome-wide level (F‑value ≥ 10.0), *significant at 5% genome-wide level (F‑value ≥ 8.10), ^†^significant at 5% chromosome-wise level (3.6 ≤ F‑value ≥ 6.0), which is assumed suggestive at the genome-wide level. pH_45_-pH value 45 min *post mortem*, pH_24_-pH value 24 h *post mortem*, meat colour_45_ L*, a*, b* meat colour traits lightness, redness, yellowness 45 min *post mortem*, meat colour_24_ L*, a*, b*- meat colour traits lightness, redness, yellowness 24 h *post mortem;* M. *long.* M. *longissimus dorsi*; M. *bic. fem.* – M. *biceps femoris*
