# Supplementary material for: A comprehensive linkage map and QTL map for carcass traits in a cross between Giant Grey and New Zealand White rabbits
Source: BMC Genet. 2015 Feb 11;16:16. doi: 10.1186/s12863-015-0168-1 (PMC4330979; doi:10.1186/s12863-015-0168-1)
Supplement: Additional file 5: Figure S2. — F value curves across OCU7 pertaining to QTL scans for carcass traits (Model 1). [file 12863_2015_168_MOESM5_ESM.docx]

## Additional file 5

## Figure S2: F value curves across OCU7 pertaining to QTL scans for carcass traits (Model1)

Marker map (Kosambi) with distances between markers in cM is given on the x-axis. The horizontal lines represent F-value thresholds at the genome-wide highly significant (α=0.01, solid) and significant (α=0.05, dashed) levels.

##
