# Supplementary material for: A comprehensive linkage map and QTL map for carcass traits in a cross between Giant Grey and New Zealand White rabbits
Source: BMC Genet. 2015 Feb 11;16:16. doi: 10.1186/s12863-015-0168-1 (PMC4330979; doi:10.1186/s12863-015-0168-1)
Supplement: Additional file 6: Table S4. — Structure of the F2 pedigree (GG x NZW). [file 12863_2015_168_MOESM6_ESM.docx]

### Additional file 6

### Table S4: Structure of the F_2_ pedigree (GG x NZW)

| Generation |  | Male | Female | $\sum$ |
| --- | --- | --- | --- | --- |
| Founder | Giant Grey (GG) | 6 |  | 6 |
|  | New Zealand White (NZW) |  | 6 | 6 |
| F_1_ |  | 9 | 33 | 42 |
| F_2_ |  | 183 | 180 | 363 |
